# Probing natural variation of *IRE1* expression and endoplasmic reticulum stress responses in Arabidopsis accessions

**DOI:** 10.1038/s41598-020-76114-1

**Published:** 2020-11-05

**Authors:** Taiaba Afrin, Minye Seok, Brenna C. Terry, Karolina M. Pajerowska-Mukhtar

**Affiliations:** Department of Biology, University of Alabama at Birmingham, 1300 University Blvd, Birmingham, AL 35294 USA

**Keywords:** Plant sciences, Natural variation in plants, Plant immunity, Plant stress responses

## Abstract

The environmental effects shape genetic changes in the individuals within plant populations, which in turn contribute to the enhanced genetic diversity of the population as a whole. Thus, individuals within the same species can acquire and accumulate genetic differences in their genomes depending on their local environment and evolutionary history. IRE1 is a universal endoplasmic reticulum (ER) stress sensor that activates an evolutionarily conserved signalling cascade in response to biotic and abiotic stresses. Here, we selected nine different Arabidopsis accessions along with the reference ecotype Columbia-0, based on their geographical origins and differential endogenous *IRE1* expression under steady-state conditions to investigate the natural variation of ER stress responses. We cloned and analysed selected upstream regulatory regions of *IRE1a* and *IRE1b*, which revealed differential levels of their inducibility. We also subjected these accessions to an array of biotic and abiotic stresses including heat, ER stress-inducing chemical tunicamycin, phytohormone salicylic acid, and pathogen infection. We measured IRE1-mediated splicing of its evolutionarily conserved downstream client as well as transcript accumulation of ER-resident chaperones and co-chaperones. Collectively, our results illustrate the expression polymorphism of a major plant stress receptor and its relationship with molecular and physiological ER stress sensitivity.

## Introduction

Because of their sessile nature, plants constantly need to respond to their surrounding environment and adapt themselves to the ever-changing conditions to ensure a suitable balance for growth and survival^1^. Thus, the native habitat imposes on species a pressure to survive and evolve along with environmental changes^2^. The multidimensional climate fluctuations trigger simultaneous genetic variations in the individuals within the plant population, contributing to an increase in the genetic diversity of the population as a whole^3^. Therefore, the individuals of the same species can exhibit distinct variation in their genome sequences depending on their geographical origins and evolutionary history. The genetic variation found in populations from different natural environments demonstrates the extent of local adaptation^4^ and allows the discovery of novel genes and alleles as signatures of plants’ adaptive responses^5^. As such, studying natural variation can provide important insights into diverse structural and functional features: novel gene and allele identification^6^, cause and effect of phenotypic variation^7^, understanding complex traits and their impact on phenotypes^8^, and selective pressure towards specific traits^8^. These discoveries can also be useful to engineer agronomically important crop plants for better compatibility with the changing climate. *Arabidopsis thaliana* (hereafter Arabidopsis) originates from continental Eurasia and North Africa but is now extensively distributed throughout the world^9^. Its natural habitat is widely diversified, from beaches to the Rocky Mountains, riverbanks to roadsides^4,9,10^. The broad spectrum of Arabidopsis natural habitats is a major contributor to its substantial genetic variation^11^. Arabidopsis natural accessions around the globe show considerable genetic and phenotypic variation in terms of plant development, physiology, and adaptation to biotic as well as abiotic stresses, manifested through traits such as ﻿rosette diameter^12^, plant height^13^, number of lateral branches^13^, leaf shape^12^, flowering time^14^, the structure of inflorescence^15^, seed dormancy^16^, drought resistance^6^, heat tolerance^17^, cold tolerance^18^, salt tolerance^11^, disease resistance^19^, resistance and tolerance to herbivores^20^, and circadian rhythms^21^. Owing to its short life cycle and small, fully sequenced genome, Arabidopsis has been at the forefront of plant model systems for the last 35 years^22^. Its worldwide distribution^9,23^, rich genetic resources^9,23^, feasibility to maintain pure lines^9^, adaptive nature^23^, availability of genome-wide single nucleotide polymorphism (SNP)^4,9^, and collections of materials developed by the international community^16^ further increased its usefulness as the model plant. The analysis of Arabidopsis natural variation has the potential to equip us with a unique understanding of functional, ecological and evolutionary connections and reationships^8^. In 1996, the Arabidopsis Genome Initiative (AGI), an international collaborative community, initiated a project to sequence the Arabidopsis genome^24^ and in 2008 followed up with a large-scale effort known as the “1001 Genome Project” to provide more refined genetic tools to the Arabidopsis community^25^. For instance, a comparative study reported that the two most-used and highly related^22^ Arabidopsis strains, Columbia-0 (Col-0) and Landsberg *erecta* (L*er*) differ by a total 25,274 SNPs in their coding and non-coding regions^24^, which underscores the abundance of genetic natural variation within Arabidopsis accessions.

The endoplasmic reticulum (ER), the largest membrane system in eukaryotic cells, plays crucial roles in a variety of cellular processes, i.e. synthesis of membrane proteins, membrane lipids, secretory proteins, protein folding, glycosylation, disulfide bonding, post-translational modifications, and packaging to target location^26,27^. Disturbances or malfunctions in any of these processes result in the accumulation of malfolded and/or unassembled proteins and subsequently trigger ER stress. The mechanisms of ER stress signalling have been studied extensively in yeast^28^, mammals^27,28^, and plants^29–31^. In plants, ER stress can be established by application of specific treatments, i.e. chemicals (tunicamycin; Tm^32,33^, dithiothreitol; DTT^31,34^, salicylic acid; SA^33,35^, L-azetidine-2-carboxylic acid^36^, cyclopiazonic acid; CPA^33^), viral and bacterial pathogens^33,37,38^, heat stress^35,39,40^, and salt stress^41^, as well as during normal growth and developmental process^31,37,42^. ER stress elicits several cellular responses, with the unfolded protein response (UPR) playing the predominant role^32,43^. UPR is a complex eukaryotic signalling pathway that functions to restore cellular homeostasis^28–30^. The key UPR signal activator and ER stress sensor, Inositol-Requiring Enzyme 1 (IRE1), is evolutionarily ancient and highly conserved in eukaryotes^43^. In Arabidopsis, two homologues of IRE1, known as IRE1a and IRE1b, are the critical players in the UPR signalling pathway^32,33,44^. *IRE1a* and *IRE1b* genes share 41% nucleotide sequence similarity and exhibit both overlapping and distinct expression patterns^32,44,45^. While both of these isoforms are expressed throughout the plant under steady-state conditions, IRE1b is specifically enriched in embryos and seeds^32,44^ and was reported to be essential for functional male fertility^46^. Under stress-induced conditions, however, the two homologues show more profound functional divergence. IRE1a plays a predominant role in biotic stresses^33^, while IRE1b is critical during abiotic stresses^39^, pointing towards genetic and physiological specialisation and diversification of the two IRE1 isoforms. How the IRE1 homologues were shaped by the evolutionary forces in diverse Arabidopsis accessions to mitigate the ER stress is an intriguing question.

Structurally, the IRE1 proteins possess well-conserved serine/threonine protein kinase and endoribonuclease (RNase) domains, which allow IRE1 to perform dual functions^47^. After sensing ER stress, IRE1 dimerises, undergoes trans-autophosphorylation, and transduces downstream UPR signalling. In Arabidopsis, IRE1a and IRE1b recognise splice-site motifs in the transcript of an evolutionarily conversed basic leucine zipper transcription factor *bZIP60* and catalyse an unconventional cytoplasmic mRNA cleavage. This processed (spliced) form of *bZIP60* mRNA undergoes translation, producing an active protein that translocates to the nucleus and transcriptionally regulates an array of UPR-responsive genes to exert a cytoprotective function^31,38,48^. The IRE1/bZIP60 signalling pathway plays a distinct role in mitigating both biotic and abiotic stresses to restore cellular homeostasis^33,49^. This unconventional splicing is referred to as regulated IRE1-dependent splicing (RIDS). Under acute or prolonged ER stress, IRE1 also degrades mRNAs through a site-specific cleavage process termed as regulated-IRE1 dependent RNA decay (RIDD)^50,51^. In Arabidopsis, RIDD mainly targets the mRNAs encoding secreted proteins^52^.

Abiotic and biotic stress factors have been shown to activate the ER stress signalling in plants. Among several environmental stressors, heat has been previously identified as a major factor affecting the vegetative and reproductive growth of plants^53,54^. Heat is also known to be a powerful inducer of UPR in yeast^55^, mammals^56^, and plants^33,35,39,40,57,58^. Upon heat stress, plant cells initiate a cascade of stress responses, including the UPR signalling in the ER^55,56^. The phytohormone salicylic acid (SA) plays a pivotal role in several growth and developmental processes^59,60^, disease resistance signaling^61,62^, and defence responses against biotrophic pathogens^63,64^. Moreover, SA was previously shown to activate the IRE1/bZIP60 arm of the UPR signalling pathway via* bZIP60* splicing^58,65,66^. Here, we selected 10 representative natural accessions of Arabidopsis and studied the contributions of genetic variation of *IRE1a* and *IRE1b* and downstream ER stress responses in the context of biotic and abiotic triggers. We showed that both *IRE1a* and *IRE1b* vary in their expression levels in the set of selected Arabidopsis ecotypes, and we demonstrated differential levels of *bZIP60* splicing in diverse ecotypes in response to SA and heat. We also detected varied accumulation levels of UPR chaperons and co-chaperons upon SA treatment. Finally, we evaluated whole-plant tolerance of the accessions to ER stress triggered by heat and Tm as well as disease resistance phenotypes of these accessions upon infection with a bacterial pathogen. Overall, we provided insights into the natural variation of ER stress responses in Arabidopsis.

## Results and discussion

### Selection of the representative accessions to study genetic variation of *IRE1a* and *IRE1b* expression

**Figure 1:**
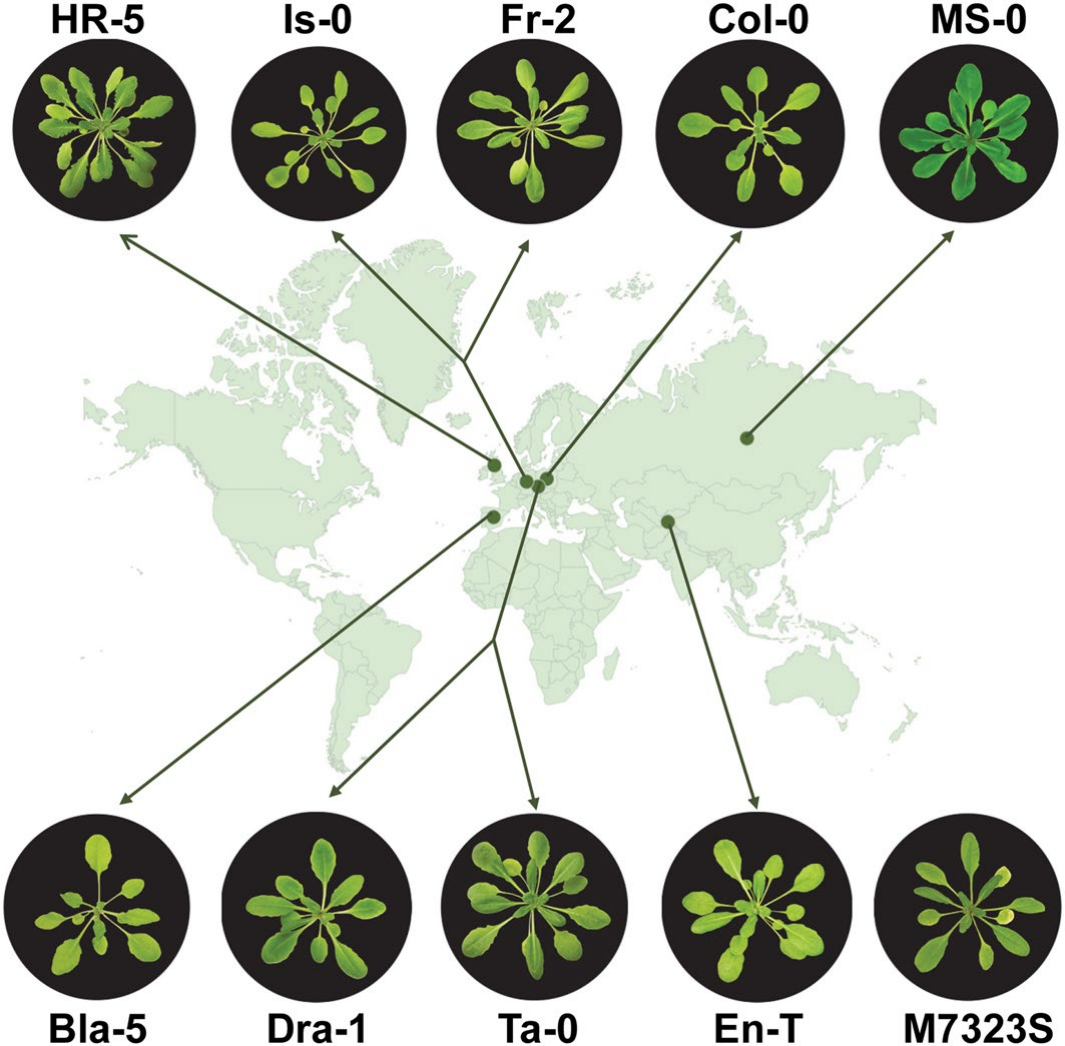
Geographical distribution and representative phenotypes of selected Arabidopsis natural accessions used in this study. Geographical origins are indicated by green dots on the world map. The origin of M7323S is unknown. 1-month-old plants representative of ecotypes Col-0, Bla-5, Dra-1, En-T, Is-0, MS-0, M7323S, HR-5, Fr-2, and Ta-0 are pictured. Plants representing individual accessions were photographed by NIKON D5600 camera. A world map background was generated using Google Sheets (a free resource). The figure was finalized using Adobe Photoshop (Version: 21.2.4).

To better understand how the evolutionary forces and natural selection have shaped the regulatory regions of *IRE1a* and *IRE1b* loci in Arabidopsis, we studied Col-0 as the reference accession along with nine additional accessions stemming from six different countries (Fig. 1, Table 1). Our selection was based on the values of basal expression levels of *IRE1a* and/or *IRE1b* genes in respective accessions, derived from the Plant eFP browser^67–70^. Specifically, we selected Bla-5, Dra-1 and En-T as accessions that display the highest basal expression of *IRE1a* compared to Col-0, while Is-0 and M7323S were the additional two accessions with the lowest basal *IRE1a* transcript levels (Table 1). We used the same strategy for *IRE1b* and selected M7323S, HR-5, and Fr-2 that exhibited elevated basal expression levels, and MS-0 and Ta-0 that were characterised by diminished basal *IRE1b* transcript accumulation compared to Col-0.

**Table 1:** Selected accessions for *IRE1a* and *IRE1b* genes, characterised in this study. Basal expression log2 ratios and fold changes (derived from Arabidopsis eFP browser) as well as geographical origin, if known, are shown.

Accessions for *IRE1a*	log2 Ratio	Fold Change	Country of Origin	Accessions for *IRE1b*	log2 Ratio	Fold Change	Country of Origin
Col-0	0.0	1.0	NW Poland	Col-0	0.0	1.0	NW Poland
Bla-5	0.7	1.62	Blanes, Spain	MS-0	0.32	0.8	Moscow, Russia
Dra-1	0.79	1.73	Drahonin, Czech Republic	M7323S	0.34	1.27	Unknown
En-T	1.12	2.17	Tajikistan	HR-5	0.29	1.22	United Kingdom
Is-0	-0.86	0.55	Isenberg, Germany	Fr-2	0.46	1.38	Frankfurt, Germany
M7323S	-0.81	0.57	Unknown	Ta-0	-0.32	0.8	Tabor, Czech Republic

### Differential response of *IRE1a* and *IRE1b* genes to heat stress

**Figure 2:**
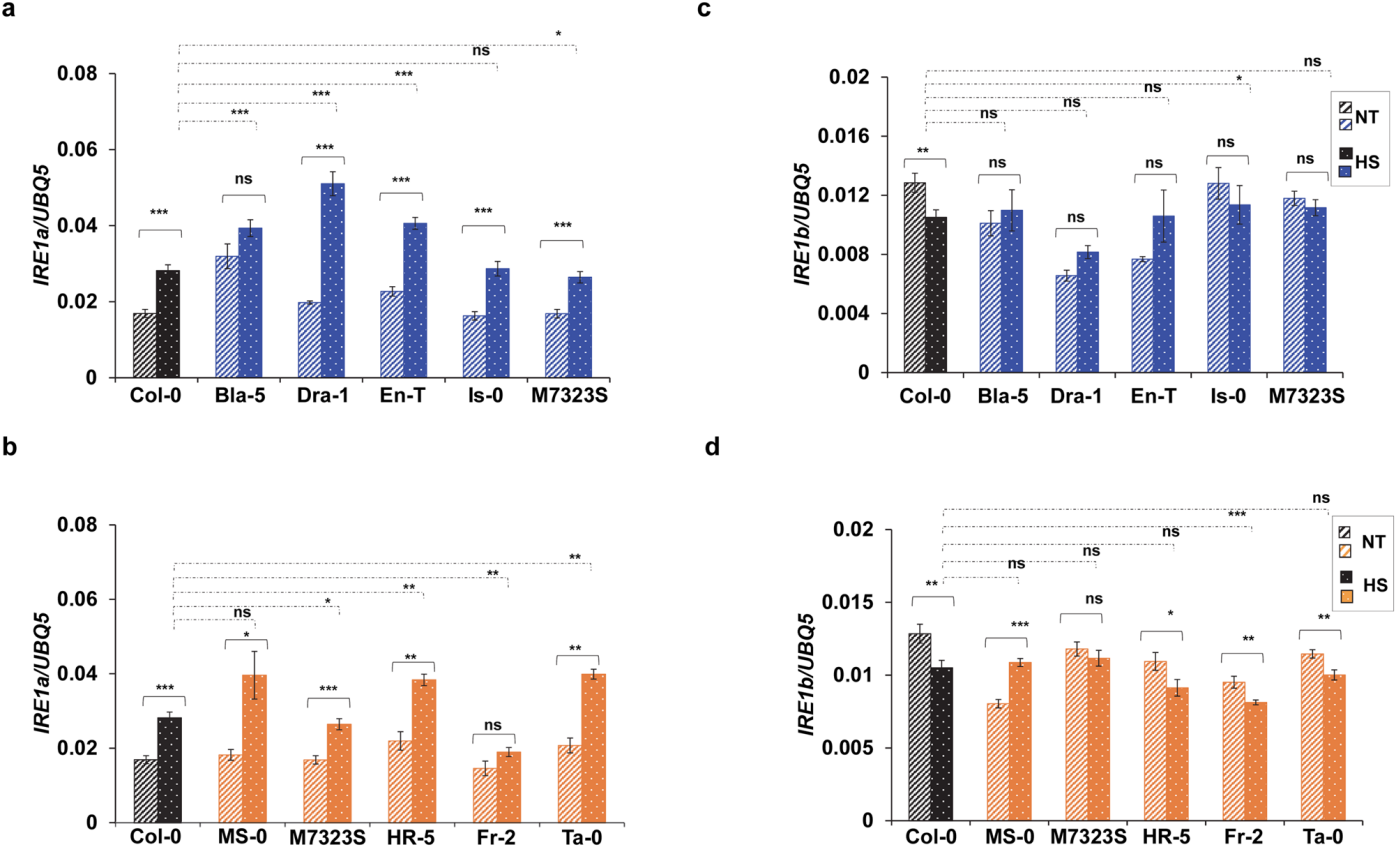
Analysis of relative *IRE1a* and *IRE1b* expression levels in selected accessions before and after heat stress. Basal and induced mRNA expression level of *IRE1a* **(a,b)** or *IRE1b* **(c,d)** of indicated accessions upon heat stress at 37ºC for 90 min. Expression levels were measured in leaf tissue of 1-month-old Arabidopsis plants via qRT-PCR and were normalised to the housekeeping gene *UBQ5* (Ubiquitin 5). Dashed bars represent basal expression levels, dotted bars correspond to heat-induced expression levels. Treatment groups are represented according to legends. Colours indicate accessions grouping (blue—IRE1a-related accessions, orange—IRE1b-related accessions, black—reference accession Col-0). Statistical analyses were performed in Excel by One-Way ANOVA. At least three independent biological replicates, each with three technical replicates were performed. Error bars show mean ± SD. Significant differences are indicated by asterisks (*** p < 0.001, ** p < 0.01, * p < 0.05), while “ns” indicates no statistically significant differences. Solid lines connecting bars represent the comparison of basal to heat-induced expression levels for each individual accession, while dashed lines represent the comparison of induced expression levels between Col-0 and an indicated accession.

We set out to validate the basal expression values obtained from the Plant eFP browser through an independent experiment. In plants, heat is known to be a powerful inducer of UPR^33,35,39,57,58^; thus, we also examined the inducibility of *IRE1a* and *IRE1b* expression following heat treatment to assess whether basal levels coincide with induced transcript accumulation in both IRE1a-and IRE1b-related groups. Towards this, we exposed our selected natural accessions to a 90-min long treatment of elevated temperature (37 °C). Following heat stress, we collected foliar tissues and quantified both basal and induced levels of *IRE1a* or *IRE1b* (Fig. 2a–d). We used an *ire1a-2 ire1b-4* double mutant as a negative control in our experiment (Fig. S1a). Our results indicated that the accessions in IRE1a-related group (Bla-5, Dra-1, En-T, Is-0, M7323S) and IRE1b-related group (MS-0, M7323S, HR-5, Fr-2, and Ta-0) showed basal *IRE1a* transcript accumulation levels comparable to the results reported by the plant eFP browser^68^ dataset (Fig. 2a,b). Specifically, Bla-5, Dra-1, and En-T plants in the IRE1a-related group exhibited higher basal *IRE1a* transcript levels, whereas Is-0 and M7323S displayed lower basal *IRE1a* accumulation. In the IRE1b-related group, we did not observe any significant change in the *IRE1a* basal transcript accumulation, consistent with the eFP browser^68^ dataset (Fig. 2b). When we analysed heat-induced *IRE1a* transcript levels in the IRE1a accession group (Fig. 2a), all ecotypes but Bla-5 significantly induced the *IRE1a* expression with respect to their basal levels. Subsequently, we compared the *IRE1a* induction in these ecotypes with the reference accession Col-0. We demonstrated that three ecotypes (Bla-5, Dra-1, and En-T) showed significantly higher *IRE1a* expression levels compared to Col-0, M7323S had a significantly reduced *IRE1a* expression, while the heat-induced *IRE1a* expression in Is-0 was comparable to Col-0. In the IRE1b-related group, *IRE1a* expression was significantly increased in all accessions except Fr-2 when compared to their corresponding controls. We also assayed the heat-induced IRE1a transcript compared to reference accession Col-0 and demonstrated a modest but statistically significant increase in the levels of expression in all accessions under study except MS-0 (Fig. 2b). When comparing fold induction above the basal levels of each accession, the strongest inducers of *IRE1a* were Dra-1, MS-0, and Ta-0 (Fig. S1b, c). Taken together, our data indicate that all of the assayed accessions show an intact ability to induce *IRE1a* following heat stress, and the basal levels are not an accurate predictor of heat inducibility for the Arabidopsis *IRE1a* gene (Fig. 2a,b).

Next, we tested *IRE1b* mRNA levels in both IRE1a- and IRE1b-related accessions. Given that M7323S displayed differential basal levels for both *IRE1a* and *IRE1b*, we also included this ecotype in the IRE1b-related category. Overall, *IRE1b* basal and induced expression remained statistically unchanged in the IRE1a-related accession group except in the reference accession Col-0, which displayed a subtle but significant reduction in the *IRE1b* mRNA levels (Fig. 2c). By comparing heat-induced *IRE1b* expression levels in the IRE1a-accession group with Col-0, we also found no statistically significant differences except for a modest increase in the Is-0 ecotype. Subsequently, we tested the basal and heat-induced *IRE1b* mRNA levels in the IRE1b-accession group. While two ecotypes (MS-0 and Ta-0) exhibited expression patterns consistent with the values reported in the plant eFP browser, we noted that the basal expression levels for M7323S, HR-5, and Fr-2 were different under our experimental conditions (Fig. 2d). Several factors in the experimental set-ups can account for such differences, including the age of plants and light cycle (4 weeks old plants and a 12/12-h light/dark cycle vs*.* 4-day-old seedlings and continuous light in our and eFP browser datasets, respectively). After we exposed the IRE1b accession group to heat, we found that the expression of *IRE1b*, unlike *IRE1a*, does not increase after heat stress, with one accession, MS-0, being a notable exception (Fig. 2d). The decreased transcript accumulation in Col-0 is also consistent with the data from the plant eFP browser^67^. When compared to the induced levels of *IRE1b* among the IRE1b-related ecotypes, we observed a trend of higher heat-induced *IRE1b* mRNA levels in Col-0 compared to all five accessions with HR-5, Fr-2, and Ta-0 displaying statistically significant differences (Fig. 2d). Analysing fold induction above the basal levels of each accession, the strongest inducers of *IRE1b* were En-T and MS-0 (Fig. S1d, e). Collectively, we concluded that the *IRE1b* expression levels were not induced upon treatment with heat in the majority of the tested ecotypes.

### Single nucleotide polymorphisms (SNPs) within the *IRE1a* and *IRE1b* promoter regions

Our results indicate that the transcriptional control of *IRE1a* and *IRE1b* differs between the two homologues and varies vastly among the natural accessions at both basal and induced levels. To gain more insights into the genetic variation that may be responsible for the observed array of transcriptional dynamics, we sequenced the predicted *IRE1a* and *IRE1b* promoter regions of Col-0 and other accessions classified into IRE1a- or IRE1b-related groups. We performed multiple sequence alignments of promoter sequences from these selected accessions and the reference sequences of Col-0. We detected a number of SNPs in transcription factor binding sites across the *IRE1a* promoter regions of Bla-5, Dra-1, and En-T (Table 2, Fig. S2a), which were the top three accessions showing elevated basal *IRE1a* expression levels. Subsequently, we subjected these polymorphic promoter regions to computational predictions of potential binding sites for transcription factors. Our bioinformatics-aided analysis identified DNA binding with one finger (DOF), Myb-related DNA binding proteins (Golden2, ARR, Psr), and Arabidopsis homeobox protein as the potential regulators (Table 2, Fig. S2a). The three accessions selected for higher basal levels of *IRE1a* (Bla-5, Dra-1, and En-T) (Table 1) all share the presence of an SNP at position 7616536 (A->T) in the predicted binding site for Myb-related DNA binding proteins, which is unique to this set of promoters and could be one of the factors contributing to elevated basal levels in those ecotypes. All newly identified promoter sequences have been submitted to NCBI GenBank under accession numbers listed in Table 2. No SNPs were detected in the predicted transcription factor binding sites of *IRE1b* promoter regions, consistent with the previously noted lack of variability in their expression patterns before and after heat treatments.

**Table 2:** SNPs found in *IRE1a* promoter regions across different Arabidopsis accessions that correspond to predicted transcription factor (TF) binding sites. Promoter and SNP positions are based on chromosomal coordinates as listed in TAIR SequenceViewer (https://seqviewer.arabidopsis.org). Nucleotide substitutions in selected accessions are indicated. The promoter sequences newly identified through this study have been submitted to NCBI GenBank under accession numbers listed.

Element Sequence	Promoter position	Predicted binding TF	SNP position	GenBank ID
acggtataAAAGcgttt	7616364–7616380	DNA binding with one finger (DOF)	7616379 Bla-5 (G->A)	MT344169
aaaATTAttta	7616531–7616536	Myb-related DNA binding proteins (Golden2, ARR, Psr)	7616536 Bla-5, Dra-1, En-T (A->T)	MT344169, MT344170, MT344171
ttAGATccgcc	7616480–7616490	Arabidopsis homeobox protein	7616481 Dra-1, En-T (G->A)	MT344170, MT344171

### Transient expression assays to understand *IRE1a* and *IRE1b* expression patterns driven through accession-specific promoter sequences

**Figure 3:**
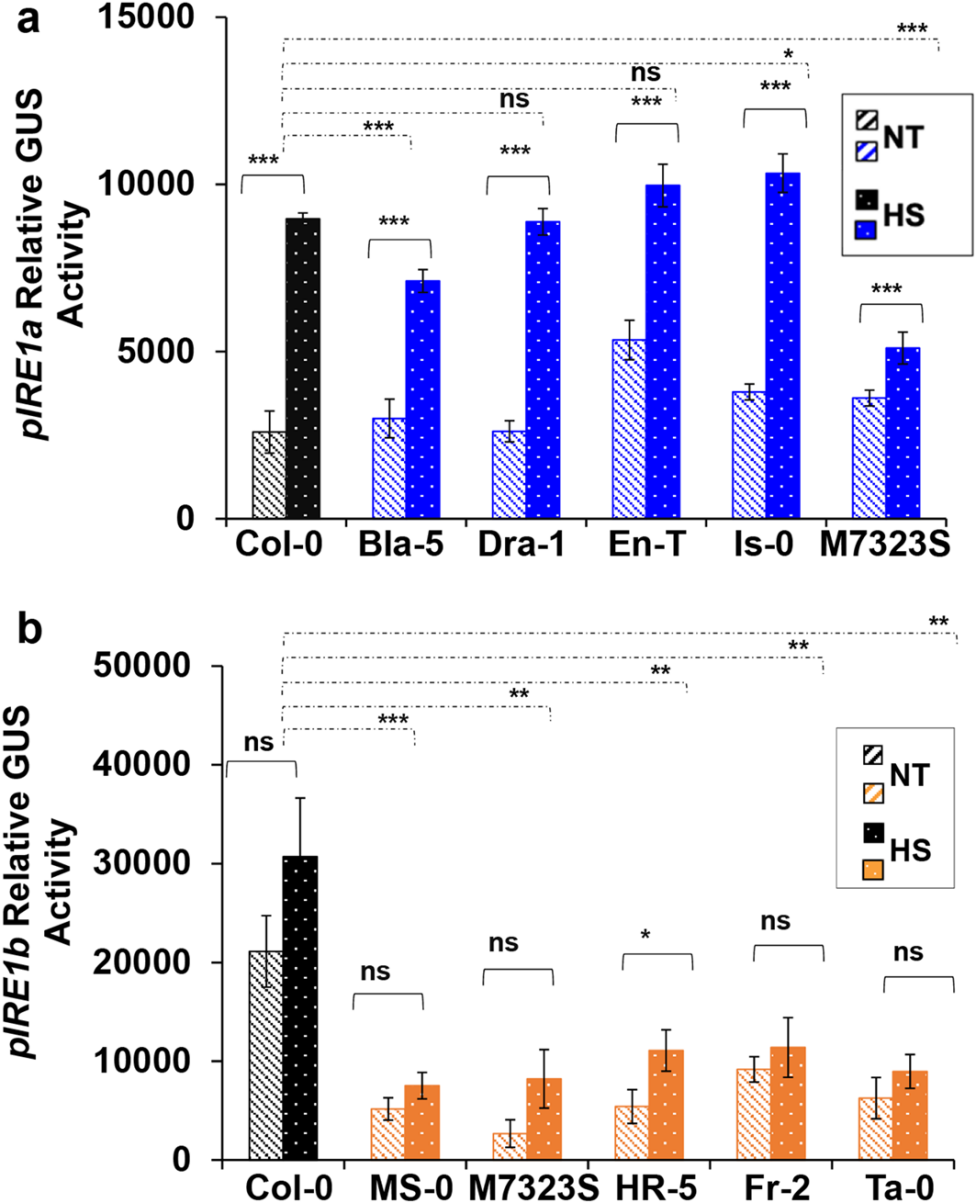
Transient MUG assay to determine basal and heat-induced activities of *IRE1a* and *IRE1b* promoters from selected ecotypes. Quantification of ß-glucuronidase (GUS) activity in Arabidopsis Col-0 leaves transiently expressing transcriptional promoter::GUS reporter fusions corresponding to *IRE1a* (pAM-PAT-pIRE1a^Col-0^-GUS, pAM-PAT-pIRE1a^Bla-5^-GUS, pAM-PAT-pIRE1a^Dra-1^-GUS, pAM-PAT-pIRE1a^En-T^-GUS, pAM-PAT-pIRE1a^Is-0^-GUS and pAM-PAT-pIRE1a^M7323S^-GUS) **(a)** and *IRE1b* (pAM-PAT-pIRE1b^Col-0^-GUS, pAM-PAT-pIRE1b^MS-0^-GUS, pAM-PAT-pIRE1b^M7323S^-GUS, pAM-PAT-pIRE1b^HR-5^-GUS, pAM-PAT-pIRE1b^Fr-2^-GUS and pAM-PAT-pIRE1b^Ta-0^-GUS) **(b)** before and after heat stress at 37ºC for 90 min. Promoter activities were determined in extracts of plant tissue via fluorometric MUG assay. Statistical analyses were performed in Excel by One-Way ANOVA. At least three independent biological replicates, each with three technical replicates were performed. Error bars show mean ± SD. Significant differences are indicated by asterisks (*** p < 0.001, ** p < 0.01, * p < 0.05), while “ns” indicates no statistically significant differences. Solid lines connecting bars represent the comparison of basal to heat-induced expression levels for each individual accession, while dashed lines represent the comparison of induced expression levels between Col-0 and an indicated accession.

To understand the potential contribution of natural variation in the regulation of *IRE1a* and *IRE1b* expression, we employed transient MUG assays. Moreover, this experiment, at least in part, provided an independent experimental method to support our qRT-PCR-based expression data (Fig. 2). Towards this, we cloned sequence-verified promoter fragments corresponding to *IRE1a* and *IRE1b* from their respective accession groups into a plant Gateway expression vector pAM-PAT-GW-GUS. This led to the generation of transcriptional promoter::GUS reporter fusions designated as pAM-PAT-pIRE1a^Col-0^-GUS, pAM-PAT-pIRE1a^Bla-5^-GUS, pAM-PAT-pIRE1a^Dra-1^-GUS, pAM-PAT-pIRE1a^En-T^-GUS, pAM-PAT-pIRE1a^Is-0^-GUS and pAM-PAT-pIRE1a^M7323S^-GUS in IRE1-related accession group. Likewise, we generated pAM-PAT-pIRE1b^Col-0^-GUS, pAM-PAT-pIRE1b^MS-0^-GUS, pAM-PAT-pIRE1b^M7323S^-GUS, pAM-PAT-pIRE1b^HR-5^-GUS, pAM-PAT-pIRE1b^Fr-2^-GUS, and pAM-PAT-pIRE1b^Ta-0^-GUS in the IRE1b-related accession group category. We transiently expressed these two sets of clones in Arabidopsis Col-0 leaves using Agrobacterium-mediated transformation over a three-day period followed by induction by heat stress at 37ºC for 90 min (Fig. 3). We collected the leaf tissues, extracted proteins, and quantified activities of ß-glucuronidase (GUS) in each sample via a fluorometric MUG assay^71^. Consistent with our qRT-PCR data, we observed a differential but significant heat-mediated induction of GUS activities driven through the set of *IRE1a* promoters compared to their respective basal levels. It is important to note that the qRT-PCR analyses were performed in the native accession backgrounds, while the MUG assays were done in Col-0 to avoid any accession-specific heterogeneity in gene regulatory mechanisms. Intriguingly, pAM-PAT-pIRE1a^Bla-5^-GUS displayed an opposite induction trend in Col-0 background (Fig. 3a) compared to its endogenous *in planta* activity (Fig. 2a). The pIRE1a^Bla-5^ sequence contains a unique SNP at nucleotide 7616379 (G->A) (Table 2), which alters a predicted binding site for a DOF transcription factor and might be one of the possible mechanisms explaining the differential inducibility of this promoter variant in the Bla-5 vs. Col-0 backgrounds. pAM-PAT-pIRE1a^Dra-1^-GUS and pAM-PAT-pIRE1a^En-T^-GUS exhibited comparable induction to pAM-PAT-pIRE1a^Col-0^-GUS, indicating that SNP 7616481 (G->A) does not cause elevated basal and/or heat-induced IRE1a promoter activity under the tested conditions. Two *pIRE1a* constructs exhibited statistically differential activity levels compared to the pIRE1a^Col-0^. While pAM-PAT-pIRE1a^Is-0^-GUS was significantly induced in Col-0 background, pAM-PAT-pIRE1a^M7323S^-GUS showed a reduced activity under the tested conditions (Fig. 3a). Overall, our results suggested that *IRE1a* promoters derived from the accessions under study exhibit differential levels of activity when tested in the reporter-based transient expression assay in Col-0 background but there is no direct correlation of heat-mediated inducibility of *pIRE1a* to the SNPs described above under our experimental conditions (Fig. 3a, Table 2).

Similarly to our results with *pIRE1a* fragments, we observed a trend of increased reporter accumulation for all *pIRE1b* constructs upon treatment with heat, although we detected a statistically significant (*p* < 0.05) expression difference only in pAM-PAT-pIRE1b^HR-5^-GUS, corroborating our qRT-PCR results (Fig. 3b). These data also further support a minor role of *IRE1b* induction under heat stress conditions. Subsequently, we compared heat-induced *IRE1b* promoter expression differences between Col-0 and the other five ecotypes. While our qRT-PCR data showed significant *IRE1b* transcript differences upon heat treatment in only three accessions (Fig. 2c,d), the MUG assay highlighted significantly increased *IRE1b* promoter::GUS activity in pAM-PAT-pIRE1b^Col-0^-GUS compared to the other five tested reporter constructs (Fig. 3b). These results indicate that the data obtained from the highly sensitive MUG assay is largely in agreement with the qRT-PCR results and further delineate the subtle *IRE1b* expression differences between Col-0 and other ecotypes. Overall, our results confirm that the heat treatment results in a more dramatic transcriptional response in the *IRE1a* expression than it is the case for its homologue, *IRE1b*. A previous study confirmed that *IRE1a* and *IRE1b* have distinct expression patterns in Arabidopsis but both can be detected in leaf tissues, biologically validating our experimental design^32^.

### Arabidopsis accessions display differential tolerance to heat and Tm-induced ER stresses

**Figure 4:**
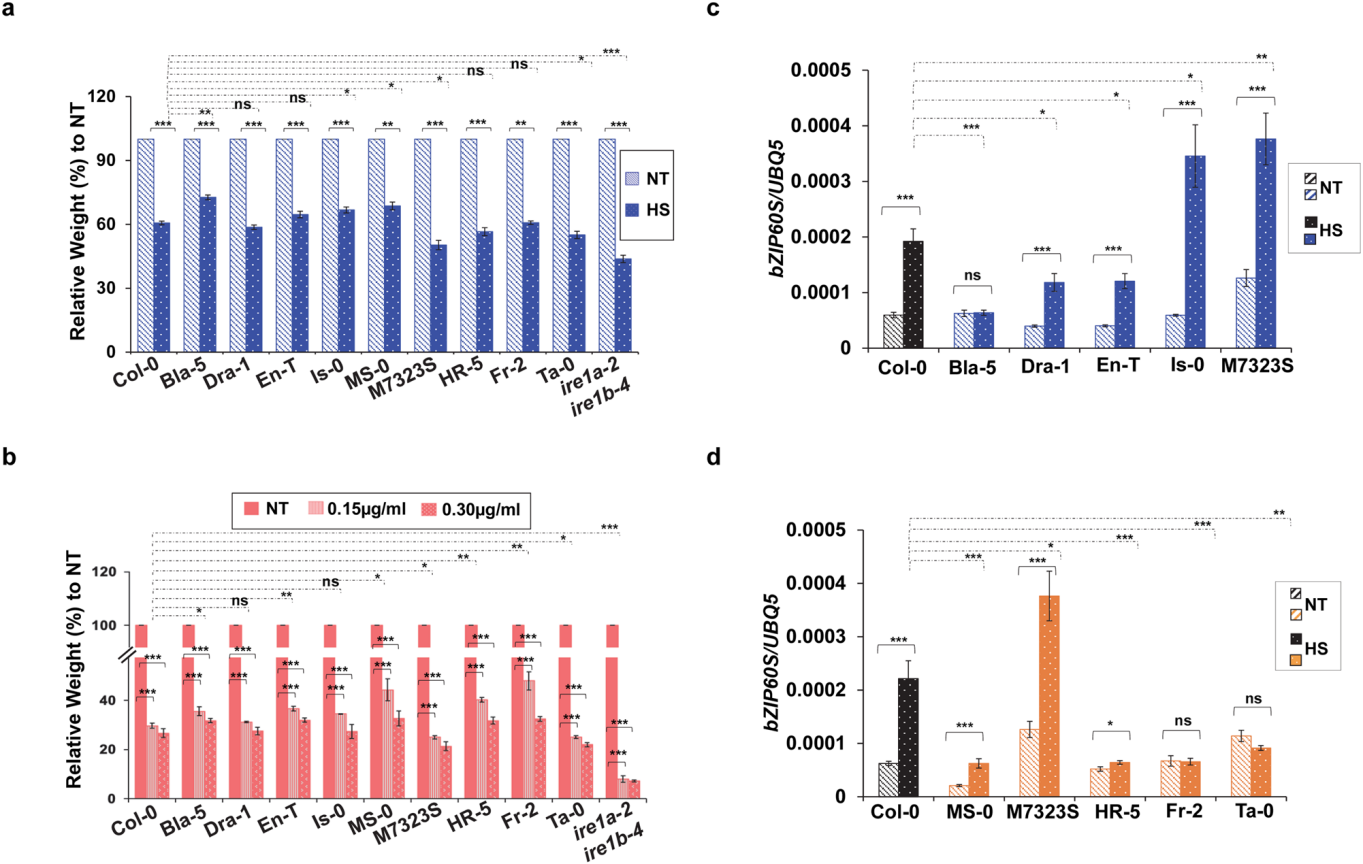
Analysis of ER stress sensitivity and relative heat-induced mRNA expression levels of spliced *bZIP60* in selected accessions. **(a)** Arabidopsis seedlings of indicated ecotypes were grown on solid half-strength MS media for 7 days, and then transferred to liquid half-strength MS media. 9-day-old plants were exposed to 42 °C for 2 h or kept at ambient temperature, and a total fresh weight of 10 plants per biological replication was recorded 2 days later. Three biological replications were performed; **(b)** 7-day-old Arabidopsis seedlings of indicated ecotypes were transferred to liquid half-strength MS media supplemented with the indicated concentration of Tm or mock. The total fresh weight of 10 plants for each of the three biological replications was recorded 3 days following Tm exposure. Statistical analyses for (a) and (b) were performed by One-Way ANOVA. Error bars show mean ± SD (n ≥ 30). Significant differences are indicated by asterisks (*** p < 0.001, ** p < 0.01, * p < 0.05), while “ns” indicates no statistically significant differences. Solid lines connecting bars represent the comparison of fresh weight between untreated and treated samples for each individual accession, while dashed lines represent the comparison of fresh weights of stress-treated plants between Col-0 and an indicated accession. Basal and induced spliced *bZIP60* (*bZIP60S*) expression levels were quantified in selected IRE1a-related accessions **(c)** or IRE1b-related accessions **(d)** upon heat stress at 37ºC for 90 min. Expression levels were measured in leaf tissue of 1-month-old Arabidopsis plants via qRT-PCR and were normalised to housekeeping gene *UBQ5* (Ubiquitin 5). Treatment groups are represented according to legends. Dashed bars represent basal expression levels, dotted bars correspond to heat-induced expression levels. Colours indicate accessions grouping (blue—IRE1a-related accessions, orange—IRE1b-related accessions, black—reference accession Col-0). Statistical analyses were performed in Excel by One-Way ANOVA. At least three independent biological replicates, each with three technical replicates were performed. Error bars show mean ± SD. Significant differences are indicated by asterisks (*** p < 0.001, ** p < 0.01, * p < 0.05), while “ns” indicates no statistically significant differences. Solid lines connecting bars represent the comparison of basal to heat-induced expression levels for each individual accession, while dashed lines represent the comparison of induced expression levels between Col-0 and an indicated accession.

To understand the potential roles of differential *IRE1a* and *IRE1b* transcript levels in different accessions, we subjected a suite of these 10 ecotypes to heat- and Tm-induced whole-plant ER stress assays. Col-0 and *ire1a-2 ire1b-4* double mutant plants were used as controls. Specifically, we exposed Arabidopsis seedlings to 42 °C for 2 h or liquid MS media supplemented with 0, 0.15 µg/mL or 0.3 µg/mL Tm, followed by total weight measurement two (heat) or three (Tm) days later. Overall, we found that all of the accessions displayed a reduction in weight in response to both ER stresses and exhibited generally consistent trends in their levels of sensitivity to ER stress caused by heat and Tm (Fig. 4a,b). In particular, the Bla-5, En-T, Is-0, MS-0, and Fr-2 accessions showed elevated tolerance to one or both types of ER stresses. In contrast, M7323S and Ta-0 were more susceptible to both ER stress-inducing treatments, while Dra-1 was not statistically different than Col-0 (Fig. 4a,b). Moreover, HR-5 displayed a somewhat divergent response, showing significantly enhanced Tm tolerance but a slight increase in heat sensitivity that was not statistically significant. The *ire1a-2 ire1b-4* double mutants showed dramatic levels of heat and Tm sensitivity, as previously described^46,72^. Consistent with the elevated basal and heat-induced expression levels of *IRE1a* in Bla-5 and En-T (Fig. 2a), we showed that these two ecotypes also displayed increased tolerance to ER stress (Fig. 4a,b). The modest improvement in the heat tolerance of Is-0 seedlings might be explained through IRE1-mediated downstream regulatory steps including bZIP60 splicing (see below) rather than its expression per se (Fig. 4c,d). Likewise, MS-0 presented a unique feature, as it is the only accession in the IRE1b-group that was characterised by increased *IRE1b* expression levels in response to heat (Fig. 2d). On the other hand, the slight increase in ER stress tolerance demonstrated by Fr-2 could also be attributed to IRE1-independent UPR signalling pathways. Finally, the diminished ER tolerance in M7323S and Ta-0 could potentially be caused by the reduced basal *IRE1b* mRNA levels in the IRE1b-accession group (Fig. 2d and Fig. 4a,b). Overall, we revealed a positive relationship between *IRE1a* and *IRE1b* expression levels or their downstream signalling activities, and observed ER stress tolerance in diverse Arabidopsis ecotypes.

### Variation in IRE1-mediated bZIP60 processing upon heat stress

A hallmark of the UPR activation in Arabidopsis is the induction of *bZIP60* mRNA splicing by IRE1a and IRE1b. Several studies previously documented a marked increase in the *bZIP60* splicing rate upon exposure to heat in Col-0^33,35,39,58^. It is important to note that the plant eFP browser does not include the transcript data corresponding to *bZIP60* splice variants. Thus, we experimentally tested the efficacy of *bZIP60* splicing in diverse ecotypes in response to heat. Regardless of the *IRE1a* or *IRE1b* expression in different ecotypes, we did not observe major differences in the basal *bZIP60* splicing efficiency when compared to Col-0. However, we did note a relatively lower *bZIP60* splicing activity in En-T, MS-0, and HR-5 (Fig. 4c,d). Subsequently, we investigated the *bZIP60* splicing efficacy under heat-induced conditions. Consistent with our previous study^43^, the reference accession Col-0 showed significant induction of bZIP60 splicing by heat (Fig. 4c,d), whereas the *ire1a-2 ire1b-4* double mutant was used as a negative control (Fig. S2b). Except for Bla-5, all the other IRE1a-related ecotypes including Dra-1, En-T, Is-0, and M7323S showed significantly increased *bZIP60* splicing, indicating a successful activation of the IRE1 signalling cascade following heat exposure. Subsequently, we measured differences in heat-induced *bZIP60* splicing across different IRE1a-related accessions. We demonstrated that the induced *bZIP60* splicing was significantly decreased in Dra-1 and En-T compared to Col-0. On the other hand, Is-0 and M7323S displayed more efficacious *bZIP60* splicing compared to Col-0 (Fig. 4c). Finally, we discerned that *bZIP60* splicing levels did not fully coincide with both basal and induced levels of *IRE1a* expression among the accessions within the IRE1a-related group (Fig. 4c). Intriguingly, we observed an inverse relationship between the induced *IRE1a* mRNA levels and *bZIP60* splicing, indicating possible existence of compensatory mechanisms between transcriptional and translational activation of IRE1a and its downstream signalling.

For the IRE1b-related accessions (Fig. 4d), we detected high levels of *bZIP60* splicing induction in Col-0, MS-0, and M7323S, and a moderate but statistically significant induction in HR-5. While *bZIP60* is a *bona fide* client for both IRE1a and IRE1b during heat stress, we observed some interdependent relationships between *IRE1* expression and *bZIP60* splicing efficiency. MS-0 was the only accession in our study that displayed enhanced *bZIP60* splicing, which is consistent with the increased levels of *IRE1b* expression following heat in that accession (Fig. 2d), and could provide a mechanistic explanation of this phenotype. M7323S plants, on the other hand, seemed to rely preferentially on the *IRE1a* transcriptional induction for bZIP60 splicing, as heat-induced *IRE1a* levels were significantly induced in that accession. Finally, the HR-5 ecotype was initially selected to be a high basal *IRE1b* expressor, but turned out to be an under-expressor with a modest induction of *bZIP60* splicing. It is possible that its ability to activate *bZIP60* splicing upon heat could be attributed to the elevated inducibility of *IRE1a* or other factors that operate at the post-translational level to regulate IRE1a/b protein activity.

Intriguingly, we did not detect any heat-induced *bZIP60* splicing in Fr-2 and Ta-0. We first confirmed that our Col-0 specific primers can hybridise to *bZIP60* orthologues from Fr-2 and Ta-0 by analysing their bZIP60 sequences provided by the 1001 Genomes Project resource^25^; henceforth, we turned to find the answers in the natural history of these two ecotypes. Both accessions originate from Northern and Central Europe (Germany and the Czech Republic), where summers are relatively short and mild, thus prolonged exposure to elevated temperatures (37 °C) might not be common in the natural habitat. Therefore, it is possible that the heat-responsive *bZIP60* splicing wasn’t shaped by the evolutionary forces in the same way as for several other accessions tested in our experiment. A previous study in mammalian kidney cells demonstrated IRE1′s downstream target *Xbp1* is spliced at 40 °C but no splicing was detected at 37 °C or 43 °C, in contrast to robust induction of *Xbp1* splicing in those cells upon treatments with DTT (inhibitor of disulfide bond formation) and thapsigargin (inhibitor of endoplasmic reticulum Ca^2+^ ATPase)^56^. These findings suggest that IRE1′s downstream splicing is precisely regulated by the temperature, and extreme heat stress may inhibit the ER stress pathway. It is possible that the heat stress of 90 min at 37 °C was perceived as acute in the Arabidopsis Fr-2 and Ta-0 accessions, and resulted in an inhibitory, instead of stimulatory, response. However, given that other accessions from the same geographical regions show intact *bZIP60* splicing ability, more work will be needed to ascertain the mechanistic basis for this observation^39^.

### Differences in IRE1-mediated UPR signalling in response to biotic stress

**Figure 5:**
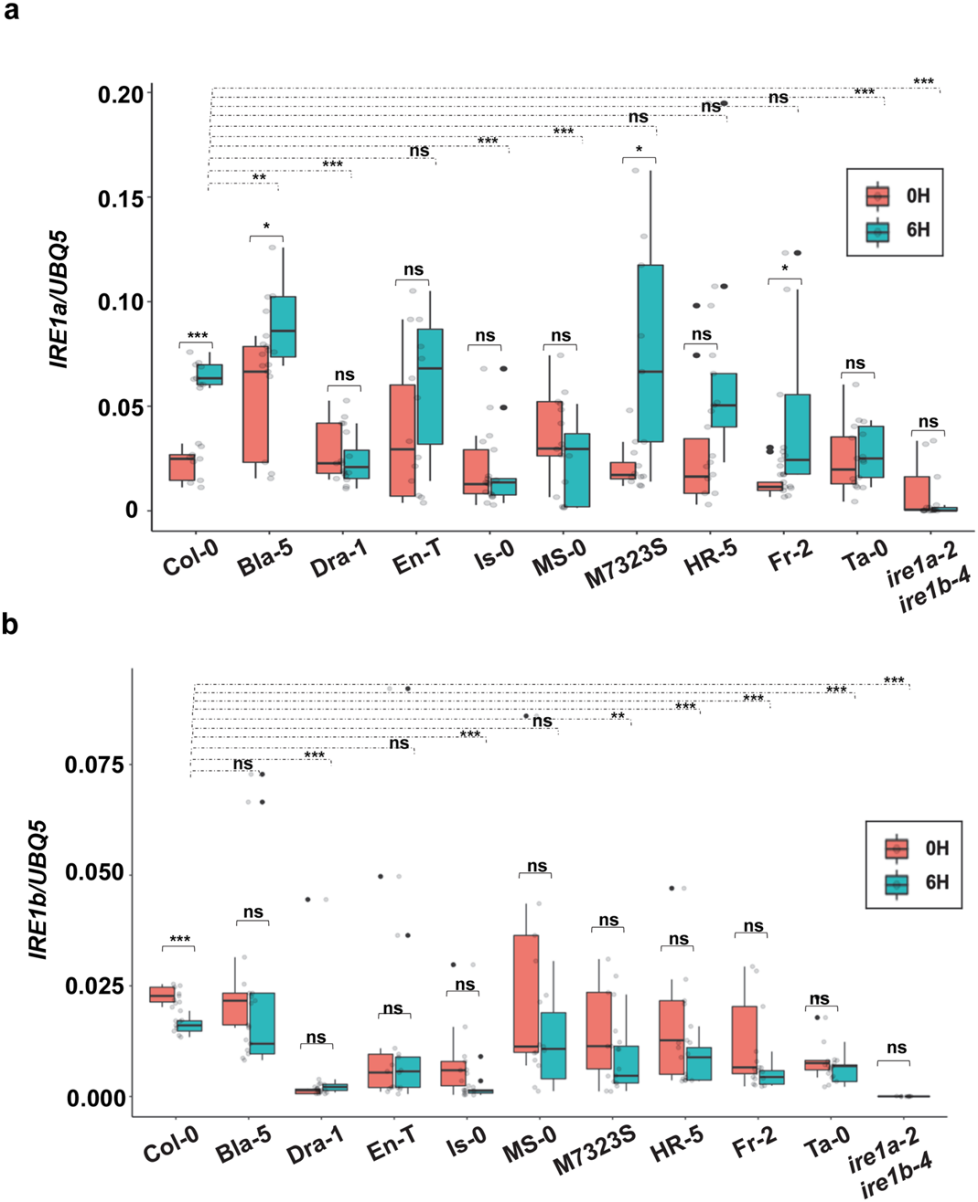
Quantification of relative mRNA levels of *IRE1a* and *IRE1b* following Salicylic Acid treatment. Transcript levels of *IRE1a* **(a)** and *IRE1b* **(b)** were quantified using qRT-PCR in leaf tissues of 1-month-old plants that were treated with 0.5 mM SA or H_2_O (mock) for 6 h. Treatment groups are represented according to legends. All expression levels were normalised to the housekeeping gene *UBQ5* (Ubiquitin 5). The box plots extend from the 25th to 75th percentiles and the whiskers extend from the minimum to the maximum levels. Light grey dots represent individual data points. Outliers, shown as dark grey dots, were identified by the test statistics of the geom_boxplot function in ggplot2. Median values were plotted in the boxes with the data generated from three independent biological replicates. Statistical analyses were performed in Excel by one-way ANOVA. Significant differences are indicated by asterisks (*** p < 0.001, ** p < 0.01, * p < 0.05), while “ns” indicates no statistically significant differences. Solid lines connecting bars represent the comparison of basal to SA-induced expression levels for each individual accession, while dashed lines represent the comparison of SA-induced expression levels between Col-0 and an indicated accession.

Given the central role of SA signalling in inducing UPR in Arabidopsis, we next set out to assess whether SA exerts differential effects on *IRE1a* and *IRE1b* mRNA levels as well as their downstream UPR signalling activities in our experimental set of Arabidopsis ecotypes. Four-week-old leaves were sprayed with 0.5 mM SA for 6 h, followed by the quantification of basal and SA-induced transcript abundance for *IRE1a* and *IRE1b* in both IRE1a- and IRE1b-group accessions. As expected, IRE1a expression was significantly induced in the reference Col-0, while no change in mRNA levels was detected in the control *ire1a-2 ire1b-4* double mutant. SA-induced *IRE1a* transcript was found to be elevated only in the Bla-5, M7323S, and Fr-2 ecotypes, while no significant change was observed in the remaining members of the IRE1a- and IRE1b-accession groups (Fig. 5a). By comparing the induced levels of *IRE1a* with those of Col-0, three accessions, namely Bla-5, M7323S, and Fr-2 exhibited significantly higher expression of *IRE1a*, whereas Dra-1, Is-0 and MS-0 displayed lower, albeit not statistically significant *IRE1a* mRNA abundance (Fig. 5a). Interestingly, Dra-1 showed an opposite regulation of *IRE1a* transcripts when exposed to two diverse ER inducing stressors, heat, and SA, whereas Bla-5 and M7323S consistently displayed the upregulation of *IRE1a* under both biotic and abiotic ER stress conditions (Fig. 2a,b, and 5a). When comparing fold induction above the basal levels of each accession, the strongest inducers of *IRE1a* were M7323S, Fr-2, Col-0, and HR-5 (Fig. S2c). In agreement with our results indicating the significant reduction of *IRE1b* transcript in response to heat, we also observed a markedly decreased *IRE1b* mRNA levels in SA-treated Col-0 plants, while no difference in the transcript abundance was observed in the control *ire1a-2 ire1b-4* double mutant (Fig. 5b). Similar to the results obtained for heat-induced *IRE1b* transcript abundance, no significant change was observed in both IRE1a and IRE1b accession groups (Fig. 5b). By comparing the *IRE1b* induction levels between Col-0 and other ecotypes, however, we observed an overall reduction of *IRE1b* transcript accumulation in our experimental set of accessions (Fig. 5b). When analysing fold induction above the basal levels of each accession, only En-T noticeably induced the *IRE1b* transcript, Bla-5 showed a minimal increase, and all the remaining accessions downregulated *IRE1b* expression in response to SA (Fig. S2d). It is worth noting that some of the ecotypes displayed very low levels of basal and/or induced *IRE1b* transcript, potentially masking some expression differences and limiting our ability to reach additional conclusions. Overall, we noted that *IRE1b* expression does not appear to be substantially changed in the majority of the ecotypes under both biotic and abiotic stress conditions (Fig. 2c,d, and Fig. 5b).

**Figure 6:**
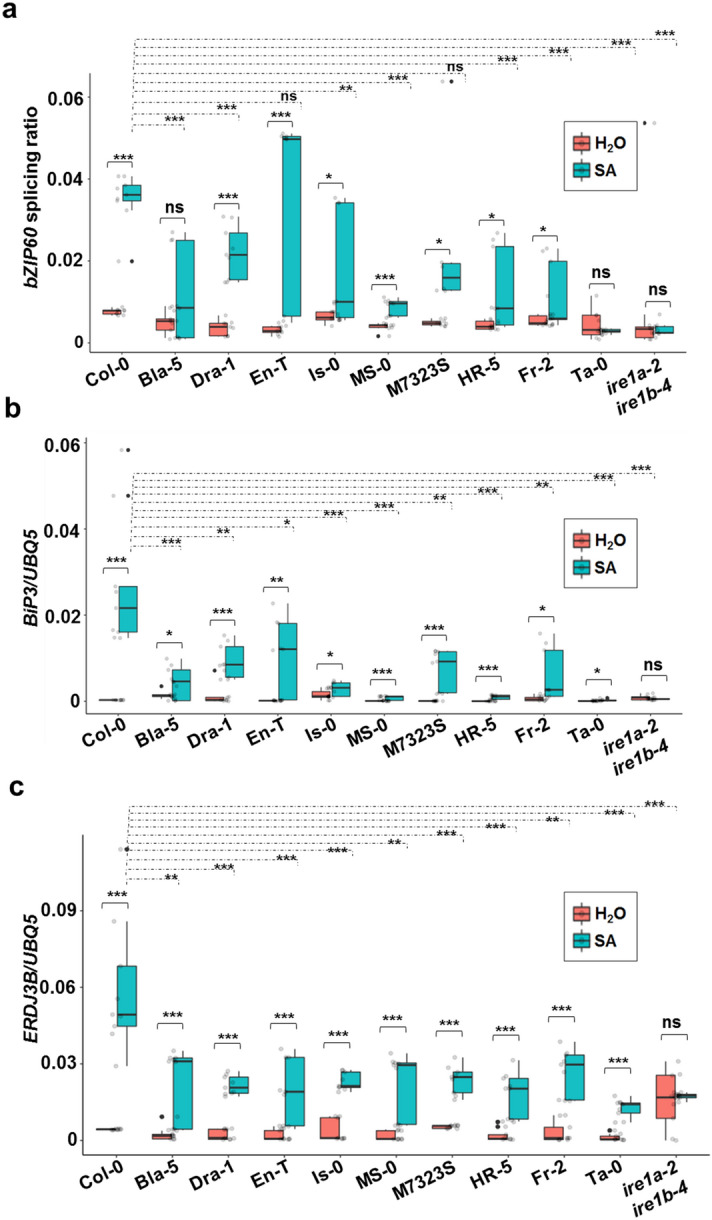
Quantification of *bZIP60* splicing efficacy and relative mRNA levels of ER stress markers *BiP3* and *ERDJ3B*. **(a)** Transcript accumulation of spliced and unspliced *bZIP60* was measured using qRT-PCR in leaf tissues of 1-month-old plants that were treated with 0.5 mM SA or H_2_O (mock) for 6 h. *bZIP60* splicing activity was calculated by normalizing values of spliced *bZIP60* to unspliced *bZIP60* transcript abundance. Transcript levels of *BiP3* **(b)** and *ERDJ3B* **(c)** were quantified using qRT-PCR in leaf tissues of 1-month-old plants that were treated with 0.5 mM SA or H_2_O (mock) for 6 h. Treatment groups are represented according to legends. All expression levels shown in panels a-c were measured in leaf tissues of 1-month-old Arabidopsis plants via qRT-PCR and were normalised to the housekeeping gene *UBQ5* (Ubiquitin 5). The box plots extend from the 25th to 75th percentiles and the whiskers extend from the minimum to the maximum levels. Light grey dots represent individual data points. Outliers, shown as dark grey dots, were identified by the test statistics of the geom_boxplot function in ggplot2. Median values were plotted in the boxes with the data generated from three independent biological replicates. Statistical analyses were performed in Excel by one-way ANOVA. Significant differences are indicated by asterisks (*** p < 0.001, ** p < 0.01, * p < 0.05), while “ns” indicates no statistically significant differences. Solid lines connecting bars represent the comparison of basal to SA-induced expression levels for each individual accession, while dashed lines represent the comparison of SA-induced expression levels between Col-0 and an indicated accession.

We next tested bZIP60 splicing in response to SA treatment. Consistent with our heat-induced splicing experiment (Fig. 4c,d), we did not observe any significant differences in basal *bZIP60* splicing activity in an independent assay (Fig. 6a), indicating the uniformity in our experimental set-up. Subsequently, we examined the efficacy of SA-induced *bZIP60* splicing in the ecotypes under study. The *bZIP60* splicing was markedly induced in the leaf tissue of the reference accession Col-0 treated with 0.5 mM SA for 6 h, while the negative control double mutant *ire1a-2 ire1b-4* did not exhibit any *bZIP60* splicing activity (Fig. 6a). Overall, despite rather low levels of transcript detected in some instances, we observed a significant induction of *bZIP60* splicing in all IRE1a- and IRE1b-related ecotypes except the Bla-5 and Ta-0 accessions. When comparing these results with heat-induced *bZIP60* splicing activity, Fr-2 was the only ecotype that exhibits a divergent response pattern to SA. Fr-2 did not activate *bZIP60* splicing under heat stress (Fig. 4d) but was capable of splicing *bZIP60* under biotic stress conditions (Fig. 6a), indicating that the ER stress induction in the Fr-2 background is more sensitised towards biotic factors. Moreover, we also observed differences in the amplitude of *bZIP60* splicing induction after SA or heat treatments indicating that individual Arabidopsis accessions can differentiate between biotic and abiotic stresses and fine-tune their ER stress responses.

In addition to *bZIP60* splicing, the UPR signalling is also manifested by the production and accumulation of various ER chaperons. Previous studies found a positive relationship of endogenous *BiP* genes expression and UPR response in Arabidopsis^31,34,38,48,73^. Luminal Binding Protein (BiP) chaperons, also known as HSP70 and GRP78, are very abundant in the ER lumen and thought to bind newly synthesised proteins as they are translocated into the ER, maintain them in a state competent for subsequent folding and oligomerisation, and prevent aggregation of malfolded proteins. *BiP1, BiP2* and *BiP3* genes are considered among the most reliable markers for ER stress regulation in plants^31,39,42,45,66,71,74^. Arabidopsis *BiP1* and *BiP2* are nearly identical in sequence, and the primers used in our analysis detect transcripts of both of those genes. Therefore, we tested the induction of *BiP1/2* and *BiP3* upon SA treatment. Except for Ta-0, all of the tested accessions showed significant upregulation of *BiP1/2* and *BiP3* expression (Fig. 6b and S3a). While displaying a statistically significant induction (*p* < 0.05), the mRNA of *BiP3* in Ta-0 was accumulated at very low levels. On the other hand, the induction of *BiP1/2* expression in this ecotype was not statistically significant. Moreover, we also observed that Col-0 was the strongest inducer of *BiP3*, while SA-mediated *BiP1/2* expression reached its highest levels in three accessions, i.e. Col-0, Bla-5, and En-T; notably, the latter two were among the high basal *IRE1a* expressors. The double mutant *ire1a-2 ire1b-4* showed a lack of significant *BiP1/2* and *BiP3* induction (Fig. 6b)^31,39,42,45,66,71,74^.

Endoplasmic reticulum dnaJ domain-containing proteins 3A and 3B (ERDJ3A and ERDJ3B) are another two molecular co-chaperones that bind to the BiP proteins in mammals and help mediate the protein folding. In Arabidopsis, ERDJ3A is responsible for functional pollen development while ERDJ3B is involved in quality control of ER proteins^75^. Both genes can be used as reliable markers for UPR activity in plants^45,73,74^. *ERDJ3A* and *ERDJ3B* both showed clear patterns of transcriptional induction following SA treatment (Fig. 6c and S3b) in all accessions with the only exception of M7323S, where *ERDJ3A* was not significantly induced after SA exposure (Fig. S3b). Similar to our findings for *BiP* genes expression, the reference accession Col-0 displayed the highest levels of *ERDJ3A* and *ERDJ3B* induction across all ecotypes tested. The double mutant *ire1a-2 ire1b-4* showed a lack of significant *ERDJ3B* induction but was able to modestly upregulate *ERDJ3A*, indicating that these two highly related chaperones have distinct transcriptional regulatory mechanisms.

In addition to BiP and ERDJ3 family members, Stromal-Derived Factor 2 (SDF2) represents another important diagnostic ER stress marker^76,77^. *SDF2* is a BTH (an SA analogue)-dependent gene^78^ and SDF2 protein can form complexes with ERDJ3B and the BiP proteins to facilitate proper ER homeostasis during PAMP triggered immunity^77^. Consistent with our findings for other ER stress markers, we observed a trend of induction in all Arabidopsis accessions tested (Fig. S3c). Bla-5 and M7323S showed the highest levels of *SDF2* induction, along with Col-0 and En-T. Ta-0 was distinguished by the lowest levels of *SDF2* transcript, and *ire1a-2 ire1b-4* double mutant showed a lack of significant *SDF2* induction. On a general note, as it was the case for the *IRE1b* expression (Fig. 5b), several ecotypes accumulated very low levels of several ER chaperone transcripts, potentially obscuring additional conclusions about the SA-mediated transcriptional regulation of those genes. Taken together, we noted differential levels of bZIP60 splicing as well as a pronounced induction of downstream UPR chaperons and co-chaperons following SA treatment in the selected natural accessions, indicating that the UPR machinery in different Arabidopsis ecotypes has evolved to cope with versatile surrounding environments.

### Response to infection with the bacterial pathogen *Pseudomonas syringae*

**Figure 7:**
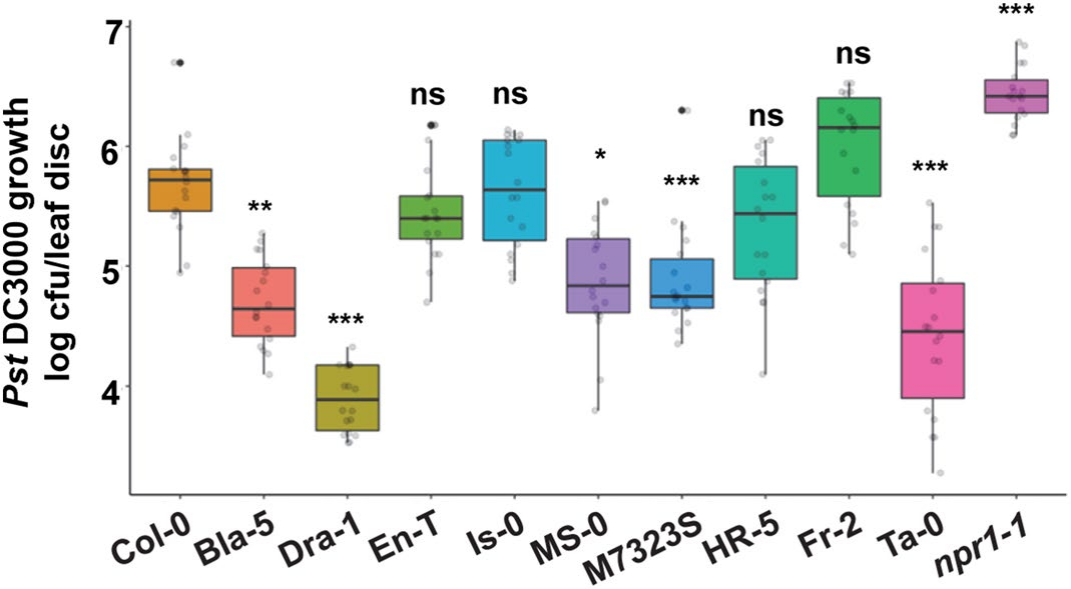
Natural variation of resistance to *Pseudomonas syringae* pv. tomato DC3000 among selected Arabidopsis accessions. Leaves of 4 weeks old plants were syringe infiltrated with *Pseudomonas syringae* pv. *tomato* strain DC3000 (*Pst* DC3000). *In planta* bacterial growth was quantified at 3 days post-inoculation. The box plots extend from 25th to 75th percentiles and whiskers extend from the minimum to the maximum levels. Light grey dots represent individual data points. Outliers, shown as dark grey dots, were identified by the test statistics of the geom_boxplot function in ggplot2. Median values were plotted in the boxes with the data generated from three independent biological replicates. Statistical analyses were performed in Excel by One-Way ANOVA. Significant differences are indicated by asterisks (*** p < 0.001, ** p < 0.01, * p < 0.05), while “ns” indicates no statistically significant differences. Black asterisks are representing the comparison of resistance or susceptibility of respective accession compared to Col-0.

We previously reported that IRE1a and IRE1b are implicated in plant immune responses to a bacterial leaf pathogen, *Pseudomonas syringae*, including basal defence and establishment of systemic acquired resistance^33^. Through a systematic genetic analysis using a suite of single and double *ire1* mutants, we previously discovered that IRE1a plays a more prominent role in mediating Arabidopsis defences against *P. syringae* than IRE1b, but both homologues exhibit some degree of functional redundancy and consequently *ire1a ire1b* double mutants display a more profound immune phenotype than the *ire1a* single mutants^33^. We hypothesised that the accessions showing higher basal and/or induced levels of *IRE1a*, *IRE1b*, SA-mediated *bZIP60* splicing, and ER-associated marker genes expression might be better equipped to fight off an infection with virulent *P. syringae* pv. *tomato* bacteria strain DC3000 (hereafter, *Pst* DC3000). To test this hypothesis, we subjected Col-0, nine natural accessions, and a hypersusceptible *npr1-1* mutant^79,80^ (negative control) to a series of bacterial infection assays^81^. The *Pst* DC3000 bacteria were pressure-infiltrated into the leaves followed by quantification of the bacterial growth three days later^81^. As expected, the *npr1-1* plants showed a susceptible phenotype with the highest bacterial loads (Fig. 7). Accessions Bla-5, Dra-1, MS-0, M7323S, and Ta-0 showed lower bacterial growth levels compared to Col-0, indicating their relative resistance to *Pst* DC3000, whereas En-T, Is-0, and HR-5 displayed a similar trend that was not, however, statistically significant. Among all ecotypes tested, Fr-2 was the only one accession that amassed slightly higher, although not statistically significant, pathogen loads when compared to Col-0 (Fig. 7). Double mutant *ire1a-2 ire1b-4* displayed significant susceptibility compared to Col-0, as reported previously^33^ (Fig. S4).

While the plant immune response is a complex process that engages numerous signalling pathways, we detected some parallels between pathogen resistance and expression of *IRE1* genes and downstream ER stress markers. Our initial hypothesis has proven correct for several accessions. For example, Dra-1, the accession with the highest basal *IRE1a* levels, showed increased heat-induced *IRE1a* expression, increased *bZIP60* splicing after the heat and SA treatments, and strong SA-mediated inducibility of *BiP1/2*, *BiP3*, *ERDJ3A*, *ERDJ3B,* and *SDF2*. Dra-5 was also the most resistant accession in our study and accumulated bacterial loads ~ 100 times lower than those of Col-0. Another resistant ecotype HR-5 (high basal *IRE1b* accession) showed reduced sensitivity to Tm, high levels of *bZIP60* splicing after the heat and SA treatments, and strong SA-mediated inducibility of *BiP1/2*, *BiP3*, *ERDJ3A*, *ERDJ3B,* and *SDF2*. Analogous conclusions can be drawn for Bla-5 (high basal IRE1a accession), which displayed a similar data trend with the exception of the *bZIP60* splicing, which was not significantly induced. Interestingly, MS-0 and M7323S are two accessions that were selected as low *IRE1a* and/or *IRE1b* expressors, yet displayed increased *bZIP60* splicing after the heat and SA treatments and substantial SA-mediated inducibility of almost all tested ER markers. This increased induction of *bZIP60* splicing and ER markers are consistent with enhanced disease resistance phenotypes of MS-0 and M7323S. On the other hand, Ta-0 is an accession that we initially selected based on reduced basal *IRE1b* transcript levels. Predictably, in our expression studies, we observed a complete lack of *bZIP60* inducibility and very low overall levels of the ER markers expression, as well as increased sensitivity to both heat and Tm. Yet, despite its poor ER-associated transcriptional signature, Ta-0 showed surprisingly low levels of *Pst* DC3000 growth, ranking as the second most resistant accession in our analysis. This finding points towards the likely existence of compensatory mechanisms, where other defence-related pathways might have been hyperactive to fend off the pathogens in their local environment while the induction of UPR machinery is impaired. In conclusion, our phytopathology analyses provided several lines of evidence for an interrelation between the relative fitness of the ER signalling pathways and overall immunity to *Pst* DC3000 infection.

### Euclidean clustering analysis reveals integrative transcriptional and phenotypic patterns

**Figure 8:**
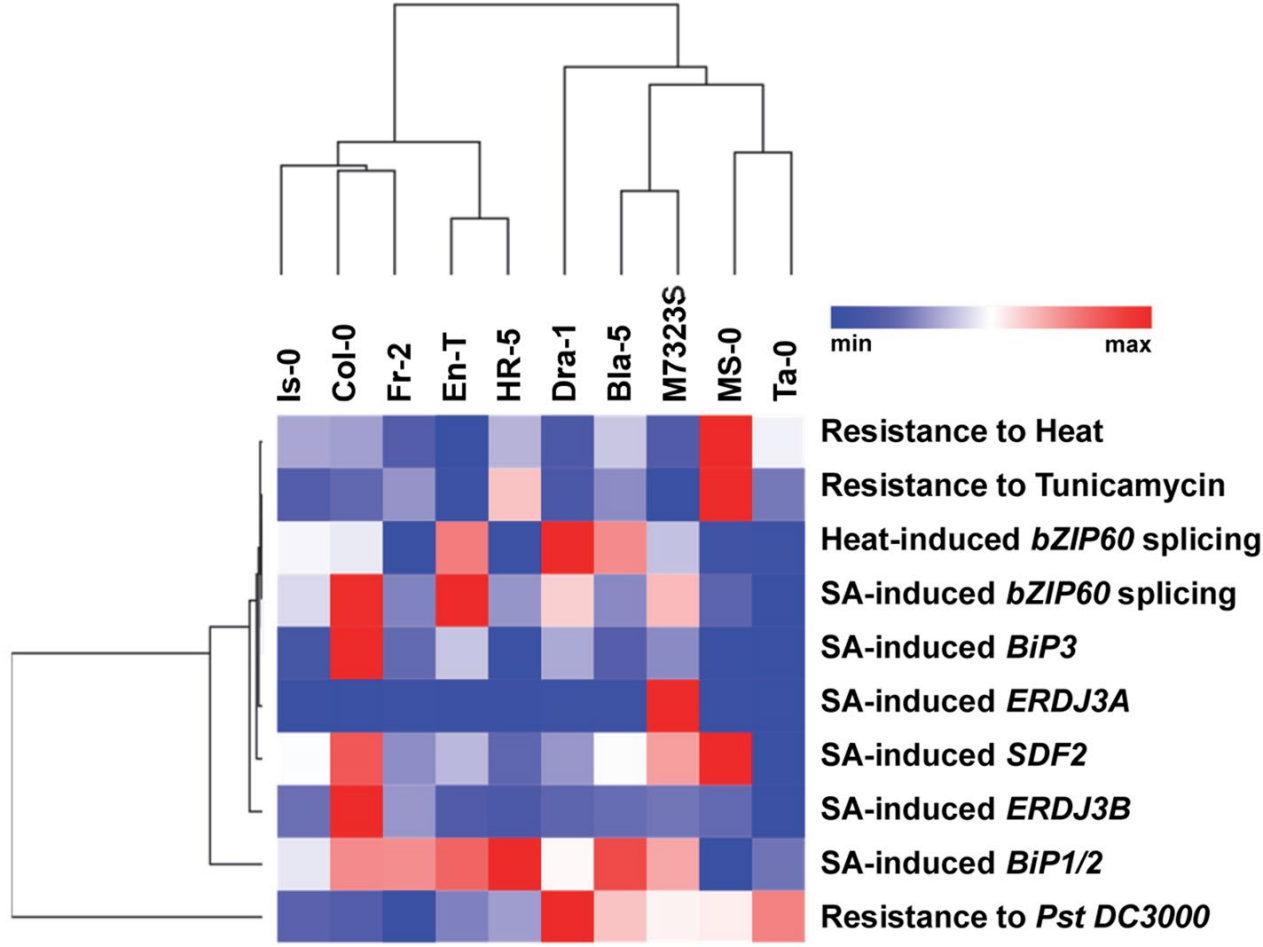
Heat map comparisons of differential gene expression, whole-plant ER stress sensitivity, and pathology phenotypes of selected Arabidopsis accessions. Responses of the selected accessions to different treatments were integrated into a heat map. Euclidean distance was used as a metric for KMeans and hierarchical clustering. In hierarchical clustering, the average was the linkage method. Accession names and treatments are indicated. Colours from red to blue indicate high to low expression/trait intensity.

To integrate our findings and uncover novel patterns and relationships between the ecotypes, we next performed Euclidean clustering analysis of the transcriptional and phenotypic responses in our panel of accessions (Fig. 8). Is-0, Fr-2 and Col-0 ecotypes share common geographical origins: Is-0 originates from Isenberg, Germany, Fr-2 stems from the neighbouring city of Frankfurt, Germany, and Col-0, often incorrectly attributed to Columbia, Missouri, USA, actually originates from north-western Poland^22^. These three accessions clustered together in our analysis, demonstrating predominantly consistent trends of stress-induced transcriptional responses, and similar levels of susceptibility to bacterial disease. MS-0 of Moscow, Russia, and Ta-0, hailing from Tabor, Czech Republic, both low basal expressors of *IRE1b*, clustered in a separate group distinguished by low amplitudes of transcriptional ER stress responses but high levels of bacterial resistance, indicating that Arabidopsis immunity to *Pst* DC3000 can be variably tied to UPR signalling depending on the specific genetic background. It is worth noting that MS-0 showed a positive relationship between high *SDF2* transcript levels and enhanced tolerance to heat and Tm, which distinguished it as the only ecotype with such phenotypic features. Interestingly, our analysis also uncovered an interrelation between the efficacy of *bZIP60* splicing and expression of *BiP3* (Fig. 6), which is consistent with the notion that *BiP3* itself has been shown to be one of the main transcriptional targets of active bZIP60 transcription factor^82^. This relationship is further reinforced by a positive feedback loop as an active bZIP60 is also able to activate its own expression through an ERSE (ER response element)-like element present in its promoter^48^. Expression trends of *BiP1/2* were independent of other markers, which is not unexpected since *BiP1* and *BiP2* are strongly and ubiquitously expressed, and weakly regulated by bZIP60^31^. *ERDJ3A*, which has been previously shown to be a heat- and bZIP60-independent ER stress marker^83^, showed expression patterns that clustered together with multiple ER chaperones following the immune stressor SA treatment. This observation is consistent with the presence of SA-inducible heat shock-like *translocon1* (*TL1) cis*-regulatory elements in *ERDJ3A* promoter^71,83^ and indicates that additional transcriptional regulators, such as TBF1^71^, might operate to bridge the UPR signalling with SA-mediated immune responses. In support of this hypothesis, TBF1 was previously shown to regulate SA-induced expression of *BiP2 *via* TL1* motifs^71^. Given the complexity of the plant immune response, it is predictable that the levels of bacterial resistance don’t show an absolute concurrence with any specific ER stress marker(s) in our analysis; however, these results prove valuable to provide interesting insights into the ecological and evolutionary relationship between the UPR and immunity to *Pst* DC3000.

## Conclusion

The genetic variation found in populations from different natural environments demonstrates the extent of local adaptation and helps gain insights into the molecular underpinnings of plant adaptive responses. This study characterised the ER stress responses in a panel of 10 natural Arabidopsis accessions and uncovered a number of variations in their UPR signatures following exposure to biotic and abiotic stress factors. Our work suggests that both Arabidopsis *IRE1* homologues, as well as their downstream signalling components, are subject to variation imposed by the evolutionary forces both at the genomic and gene regulation levels. We provided new insights into the natural diversity of a ubiquitous and evolutionarily conserved cellular stress signalling pathway, and our discoveries can form a foundation to engineer climate-resilient crop plants; a necessity for a sustainable future.

## Materials and methods

### Plant material and growth conditions

The seeds of selected *A. thaliana* accessions listed in Table 1 were ordered from the Arabidopsis Biological Resource Center (ABRC, Ohio State University, Columbus, OH, USA). All the seeds were sown on sterilised soil (SunGro Horticulture, Super-Fine Germinating Mix) in individual pots. The seeds were stratified for seven days in a cold room facility at 4 °C. The pots were then transferred to a controlled growth room facility (12 h light/12 h dark photoperiod; 21 °C; 100 μmol/m^2^/s light intensity and 40% relative humidity). 10–15 days old seedlings were then transplanted into 72-well flats for growth (1 month) and subsequent experimentation.

### Plant photography

The pictures of the plants representing individual ecotypes were taken by NIKON D5600 camera and were edited using Adobe Photoshop (Version: 21.2.4).

### Selection of accessions

The accessions (Table 1) were selected based on *IRE1a* and *IRE1b* expression patterns in Arabidopsis eFP (electronic Fluorescent Pictograph) browser, available at https://www.bar.utoronto.ca/. Logarithmic fold-change values were provided by ePLANT, with relative logarithmic values above 0.7 and below -0.8. The accessions that showed the highest extent of variation, characterised by the highest and lowest expression values for *IRE1a* and *IRE1b* were selected.

### Heat stress assays and salicylic acid treatments

1-month-old Arabidopsis plants grown in soil were exposed to heat stress at 37 °C for 90 min in an incubator, then leaf tissue was sampled. For phytohormone treatment, aerial parts of 1-month-old soil-grown Arabidopsis plants were sprayed with 0.5 mM SA, covered with a dome for 2 h, and leaf samples were collected after 6 h post-treatment. At least three leaves derived from three independent plants were collected for each ecotype/treatment/time point combination.

### ER stress assays

Seeds from different Arabidopsis accessions were washed with 70% Ethanol and 0.05% Triton and then stratified at 4 °C for 3 days on half-strength solid Murashige Skoog (MS) media plates (Phytotechnology Labs, Overland Park, KS, USA). After stratification, MS plates were transferred to growth chambers (12 h light/12 h dark photoperiod; 21 °C; 100 μmol/m^2^/s light intensity and 40% relative humidity). 0.15 μg/mL or 0.30 μg/mL of Tunicamycin (Tm) (Tocris Bioscience; 3516/10) were used as a chemical ER stressor. 7 days old seedlings were transferred to liquid half-strength MS media with or without the appropriate concentration of Tm. The total fresh weight of 10 plants for each biological replications was recorded 3 days following Tm exposure. For heat stress, Arabidopsis seedlings were grown on solid half-strength MS media for 7 days and then transferred to liquid half-strength MS media. 9 days old Arabidopsis plants were exposed to 42 °C for 2 h and the total weight was recorded 2 days later.

### mRNA quantification and bZIP60 splicing

Gene expression analysis was conducted as described previously^84^. In brief, leaf tissue from 1-month-old plants was collected at designated time points. At least three leaves derived from three independent plants were collected for each genotype/ treatment/ time point combination. Trizol reagent (Invitrogen) was used to extract total RNA and DNase I (Ambion) was applied to remove DNA contaminants. 10 μg of total RNA were reverse transcribed using SuperScript III first-strand RT-PCR kit (Invitrogen), and quantitative gene expression analysis was determined using GoTaq qPCR Master Mix (Promega) with transcript-specific primers in a RealPlex S MasterCycler (Eppendorf). The Ct values were normalised to ubiquitin 5 (*UBQ5*) gene. bZIP60 splicing assays were performed as described in Moreno et al.^33^. Briefly, we used a common forward primer and a pair of reverse primers that specifically hybridise to either the unspliced or spliced variants of cDNA originated from bZIP60 mRNA, respectively. This allows for the detection of two specific qRT-PCR products corresponding to unspliced and spliced bZIP60 variants (Fig. S5). Primers used for qRT-PCR are listed in Table S1.

### Preparation of promoter::GUS constructs

DNA extraction was performed from 1 month old plants with 200 μl CTAB extraction buffer (2% cetyl-trimethyl ammonium bromide, 100 mM tris [pH 8.0], 20 mM EDTA pH [8.0], 1.4 M NaCl, 0.5% β-Mercaptoethanol, 2% polyvinyl pyrrolidone). The promoter region of *IRE1a* (~ 1.267 kb) and *IRE1b* (~ 1.477 kb) from different accessions were amplified from genomic DNA by PCR using Phusion Polymerase (Thermo Scientific) with attB-flanked primers (Table S1). The PCR products were cloned into pDONR207 Gateway vector via BP reactions (Invitrogen). After confirming the entry clones through PCR and Sanger sequencing (primers listed in Table S1), destination clones were constructed by LR reactions with binary Gateway vector pAM-PAT-GW-GUS and confirmed through PCR and Sanger sequencing. The plant expression vector pAM-PAT-35S-GW-GUS was a gift from Drs. Nico Dissmeyer and Imre Somssich (Addgene plasmid # 80,678; https://n2t.net/addgene:80678; RRID:Addgene_80678). The resulting pAM-PAT-promoter-GUS constructs were transformed into *Agrobacterium tumefaciens* (strain GV3101) for transient expression assays.

### *IRE1a* and *IRE1b* promoter analyses

We analysed the obtained promoter sequences using 4Peaks software (https://nucleobytes.com/4peaks/index.html). The confirmed sequences were aligned with the *IRE1a* or *IRE1b* promoter sequence of the reference accession Col-0 using MultAlin website (https://multalin.toulouse.inra.fr/multalin/) (Fig. S2). The promoter sequences from Col-0 were used to predict putative transcription factor binding sites using the software MatInspector (https://www.genomatix.de/online_help/help_matinspector/matinspector_help.html) and the website PlantRegMap (https://plantregmap.gao-lab.org). The predicted TF target sequences were matched with the SNPs identified from MultAlin website. The newly identified TF binding sites with SNPs have been submitted to NCBI GenBank under the following accession numbers: MT344169, MT344170, and MT344171.

### Quantitative GUS assay

1-month-old Col-0 plants were agroinfiltrated with a needleless syringe as described previously^85^. Three days post-inoculation, the plants were exposed to heat stress in an incubator at 37 °C for 90 min. Immediately following the heat stress, the tissues were collected and ground under liquid nitrogen. Total proteins from the harvested tissue were extracted with extraction buffer (50 mM NaP0_4_ [pH 7.0], 1 mM Na_2_EDTA, 0.1% SDS, 0.1% Triton X-100, protease inhibitor for plant extracts [Sigma], and 10 mM β-mercapethanol) as described previously^71^. Followed by centrifugation (10 min, 4000×*g*, 4 °C) the supernatants were collected, and protein concentration was quantified using Bradford Reagent (Sigma). The extracted proteins were incubated with 1 mM MUG (4-methylumbelliferyl β-D-glucuronide) to quantify GUS activity. 1 M Na_2_CO_3_ was used as a stop buffer to terminate the reaction and fluorescence was measured with a microplate reader (Tecan) with an excitation wavelength of 365 nm, an emission wavelength of 455 nm and a filter wavelength of 430 nm. The relative MUG values were obtained by normalizing data to the Bradford assay. The experimental procedures were adjusted based on a previously published protocol^86^.

### Bacterial strains and bacterial growth quantification

*Pseudomonas syringae* pv. *tomato* DC3000 (*Pst* DC3000) was used for pathogen infection and quantification assay. 1-month-old soil-grown plants were syringe-infiltrated with *Pst* DC3000 (OD_600_ = 0.0002). 3 leaves/plant, 6 plants/replication, and at least three independent biological replications were performed. Bacterial growth was quantified three days of post inoculation as described previously^81^.

### Heat map and Euclidean clustering analysis

The heat map was generated using the website Morpheus (https://software.broadinstitute.org/morpheus/). Euclidean distance was used as a metric for KMeans and Hierarchical clustering. In hierarchical clustering, the average was the linkage method. Colors from red to blue indicate high to low expression/trait intensity.

### Statistical analysis

Statistical differences were calculated by one-way ANOVA in Excel and R. ggplot2 was used to make graphs shown in Figs. 5, 6, and 7, and supplemental Figs. 3 and 4. Statistically significant differences are indicated with *p < 0.05, **p < 0.01, ***p < 0.001, and ****p < 0.0001.

### Creative commons

Natural Variation of IRE1 in Arabidopsis” by Taiaba Afrin is licensed under CC BY-SA 4.0. To view a copy of this license, visit https://creativecommons.org/licenses/by-sa/4.0.

## Data and materials availability

All data needed to evaluate the conclusions in this article are present in the paper and/or the Supplementary Materials. Additional data related to this paper may be requested from the authors. The reported mutant seeds and plasmids can be provided by KPM pending scientific review and a completed material transfer agreement.

## Supplementary information

Supplementary Information
