# Supplementary material for: Probing natural variation of IRE1 expression and endoplasmic reticulum stress responses in Arabidopsis accessions
Source: Sci Rep. 2020 Nov 5;10:19154. doi: 10.1038/s41598-020-76114-1 (PMC7645728; doi:10.1038/s41598-020-76114-1)
Supplement: Supplementary file 1 — Supplementary Information [file 41598_2020_76114_MOESM1_ESM.pdf]

Supplementary File

for

Probing natural variation of *IRE1* expression and endoplasmic reticulum stress responses in Arabidopsis accessions

Taiaba Afrin, Minye Seok, Brenna C. Terry, Karolina M. Pajerowska-Mukhtar

Supplementary Figures

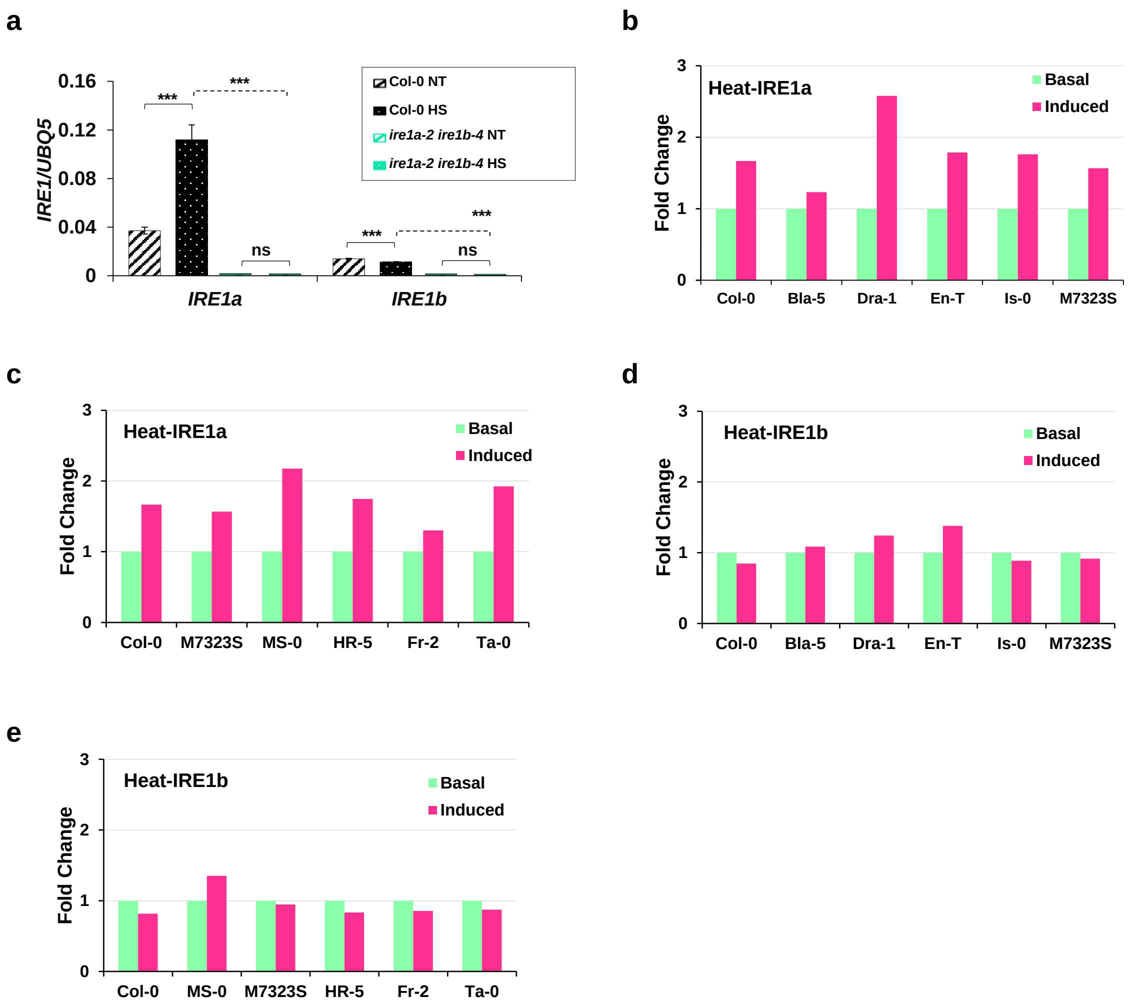

Supplementary Figure 1: Analysis of relative *IRE1a* and *IRE1b* expression levels before and after heat stress. (a) Basal and induced mRNA expression levels of *IRE1a* and *IRE1b* in

Col-0 (black bars) and double mutant *ire1a-2 ire1b-4* (green bars) were measured in leaf tissue of 1-month-old Arabidopsis plants that were untreated or heat-stressed at 37ºC for 90 minutes. Transcript accumulation was assessed *via* qRT-PCR and normalized to housekeeping gene *UBQ5* (Ubiquitin 5). Dashed bars represent basal expression levels, and dotted bars correspond to heat-induced expression levels. Statistical analyses were performed in Excel by One-Way ANOVA. At least three independent biological replicates, each with three technical replicates were performed. Error bars show mean ± SD. Significant differences are indicated by asterisks (*** $p<0.001$, ** $p<0.01$, * $p<0.05$). Solid lines connecting bars represent the comparison of basal to heat-induced expression levels for each individual genotype, while dashed lines represent the comparison of induced expression levels between Col-0 and *ire1a-2 ire1b-4* plants. **(b,c)** The fold change of induced *IRE1a* expression compared to the respective basal expression levels in the selected members of the IRE1a-accession group **(b)** and IRE1b-accession group **(c)** upon heat stress. **(d,e)** The fold change of induced IRE1b expression compared to the respective basal expression levels in the selected members of the IRE1a-accession group **(d)** and IRE1b-accession group **(e)** upon heat stress**.** The fold change was calculated by dividing the average (from 3 biological replications) of induced expression value with average (from 3 biological replications) of basal expression value. Treatment groups are represented according to legends.

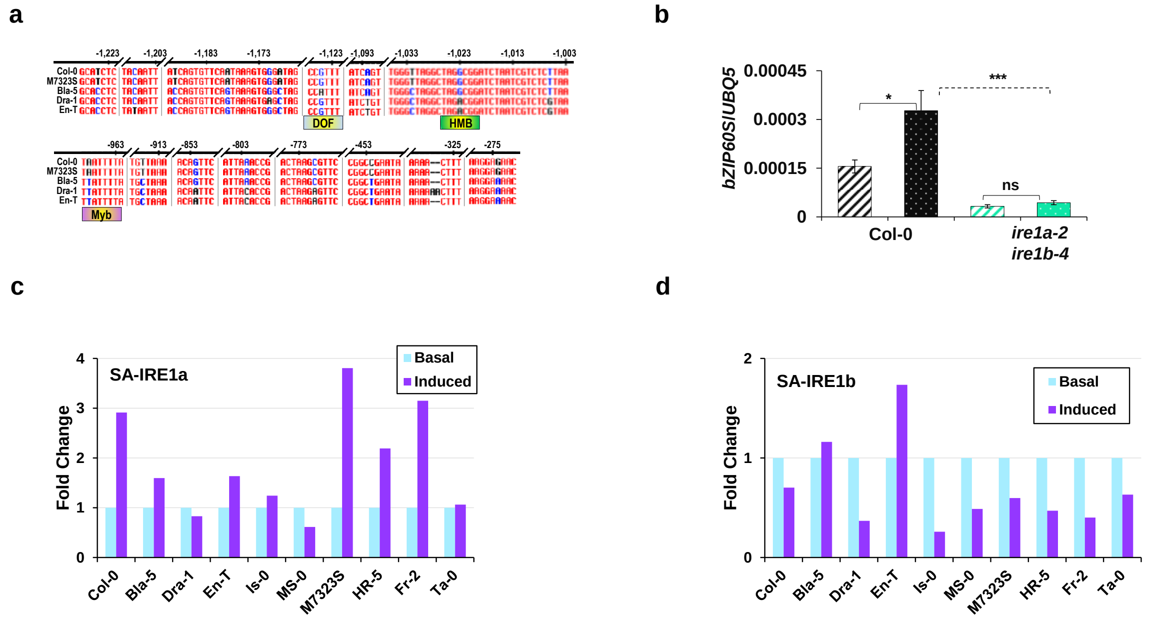

Supplementary Figure 2: (a) multiple sequence alignment of the promoter regions of the *IRE1a* gene among five ecotypes. Upstream regulatory sequences of *IRE1a* from Col-0, M7323S, Bla-5, Dra-1, and En-T were obtained and aligned to identify polymorphic regions. For clarity, only the sequence regions showing polymorphisms (17 SNPs and one InDel) among these accessions are shown. Positions are relative to the point of translation initiation coding sequence in Col-0. Predicted binding sites for transcription factors DOF (DNA binding with one finger), Myb (Myb-related DNA binding proteins), and HMB (Arabidopsis homeobox protein) are indicated with rectangular boxes. **(b)** Basal and induced mRNA expression levels of spliced bZIP60 (bZIP60s) in Col-0 (black bars) and double mutant *ire1a-2 ire1b-4* (green bars) were measured in leaf tissue of 1-month-old Arabidopsis plants that were untreated or heat-stressed at 37ºC for 90 minutes. Transcript accumulation was assessed *via* qRT-PCR and normalized to housekeeping gene *UBQ5* (Ubiquitin 5). Dashed bars represent basal expression levels, and dotted bars correspond to heat-induced expression levels. Statistical analyses were performed in Excel by One-Way ANOVA. At least three independent biological replicates, each with three technical replicates were performed. Error bars show mean ± SD. Significant differences are indicated by asterisks (*** $p<0.001$, ** $p<0.01$, * $p<0.05$). Solid lines connecting bars represent the comparison of basal to heat-induced expression levels for each individual genotype, while dashed lines represent the comparison of induced expression levels between Col-0 and *ire1a-2 ire1b-4* plants. **(c)** The fold change of induced IRE1a expression compared to the basal expression levels in all the selected accessions upon SA stress. **(d)** The fold change of induced IRE1b expression compared to the basal expression levels in all the selected accessions upon SA stress. The fold change was calculated by dividing the average (from 3 biological replications) of induced expression value with average (from 3 biological replications) of basal expression value. Treatment groups are represented according to legends.

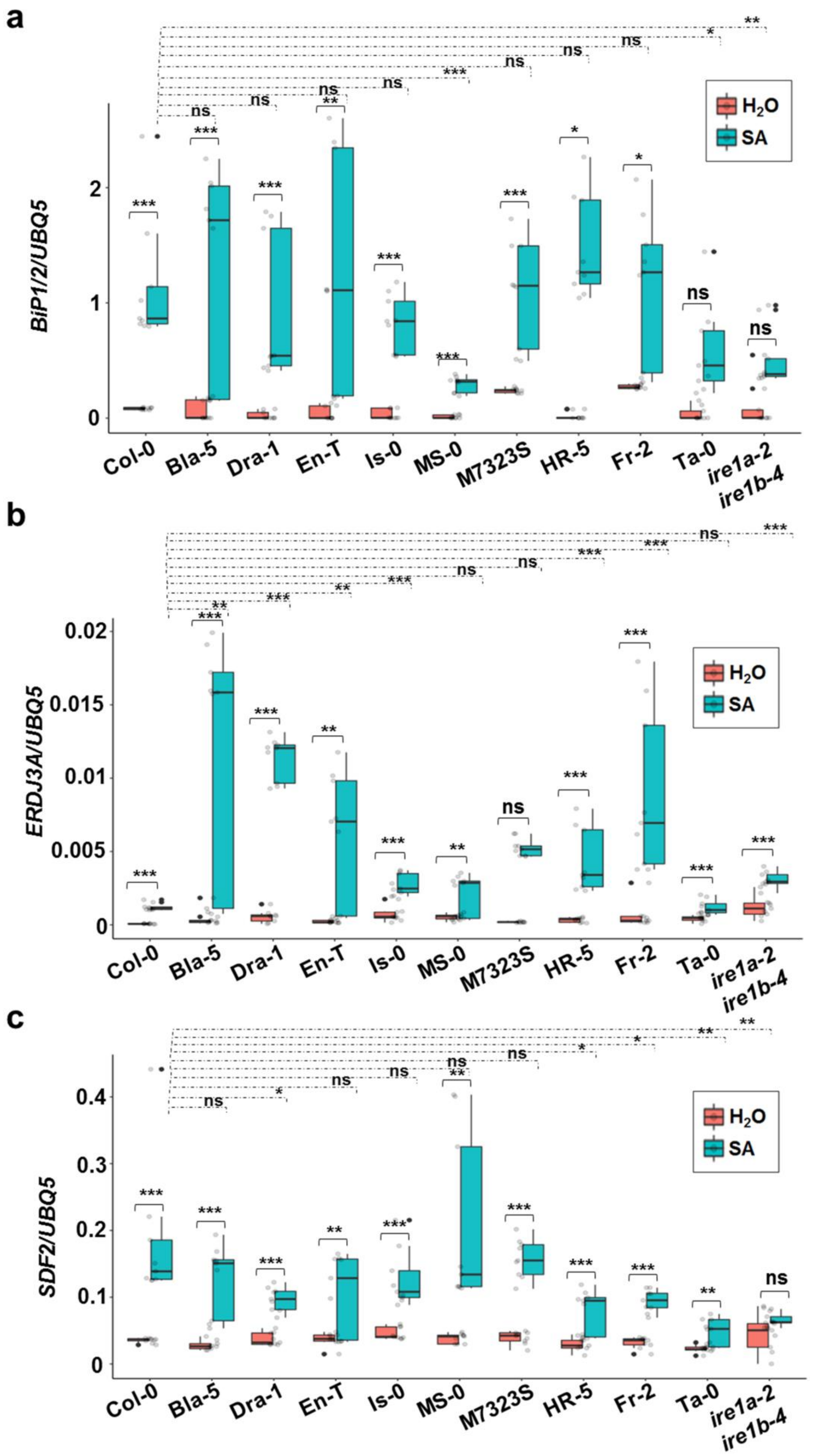

Supplementary Figure 3: Quantification of relative mRNA levels of ER stress markers *BiP1/2*, *ERDJ3A,* and *SDF2*. Transcript levels of *BiP1/2* **(a)**, *ERDJ3A* **(b),** and *SDF2* **(c)** were

quantified using qRT-PCR in leaf tissues of 1-month-old plants that were treated with 0.5mM SA or H_2O (mock) for 6 hours. Treatment groups are represented according to legends. All expression levels shown in panels a-c were measured in leaf tissues of 1-month-old Arabidopsis plants *via* qRT-PCR and were normalized to housekeeping gene *UBQ5* (Ubiquitin 5). The box plots extend from the 25th to 75th percentiles and the whiskers extend from the minimum to the maximum level. Light grey dots represent individual data points. Outliers, shown as dark grey dots, were identified by the test statistics of the geom_boxplot function in ggplot2. Median values were plotted in the boxes with the data generated from three independent biological replicates. Statistical analyses were performed in Excel by one-way ANOVA. Significant differences are indicated by asterisks (*** p<0.001, ** p<0.01, * p<0.05). Solid lines connecting bars represent the comparison of basal to SA-induced expression levels for each individual accession, while dashed lines represent the comparison of SA-induced expression levels between Col-0 and an indicated accession.

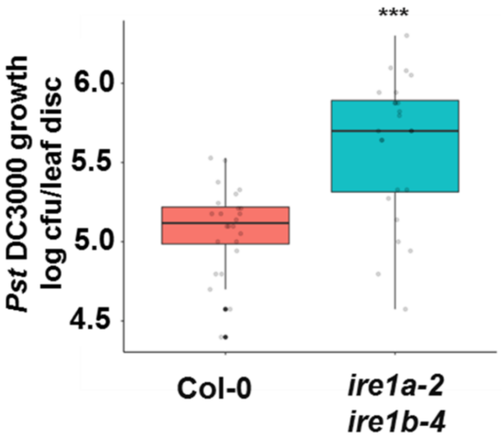

Supplementary Figure 4. Enhanced disease susceptibility to *Pseudomonas syringae* pv. tomato DC3000 in double mutant *ire1a-2 ire1b-4*.

Leaves of 4 weeks old plants were syringe infiltrated with *Pseudomonas syringae* pv. tomato DC3000 (*Pst* DC3000). *In planta* bacterial growth was quantified at 3 days post-inoculation. The box plots extend from 25th to 75th percentiles and whiskers extend from minimum to maximum level. Light grey dots represent individual data points. Outliers, shown as dark grey dots, were identified by the test statistics of the geom_boxplot function in ggplot2. Median values were plotted in the boxes with the data generated from three independent biological replicates. Statistical analyses were performed in Excel by One-Way ANOVA. Significant differences are indicated by asterisks (*** p<0.001).

Supplementary Figure 5. Schematic representation of the q-RT-PCR assay developed to measure levels of spliced and unspliced bZIP60 variants. The binding site for the common forward primer is highlighted in yellow. The binding site for the reverse primer specific to unspliced bZIP60 is highlighted in green. Sequence marked in pink and orange constitutes the binding site for the reverse primer specific to spliced bZIP60. Sequence coordinates are indicated next to Reference Sequence (top) and are in relation to the bZIP60 CDS sequence. A schematic representation of PCR amplicon sizes is shown to the right. The unspliced bZIP60 fragment is 270bp while the spliced bZIP60 amplicon is 262bp.

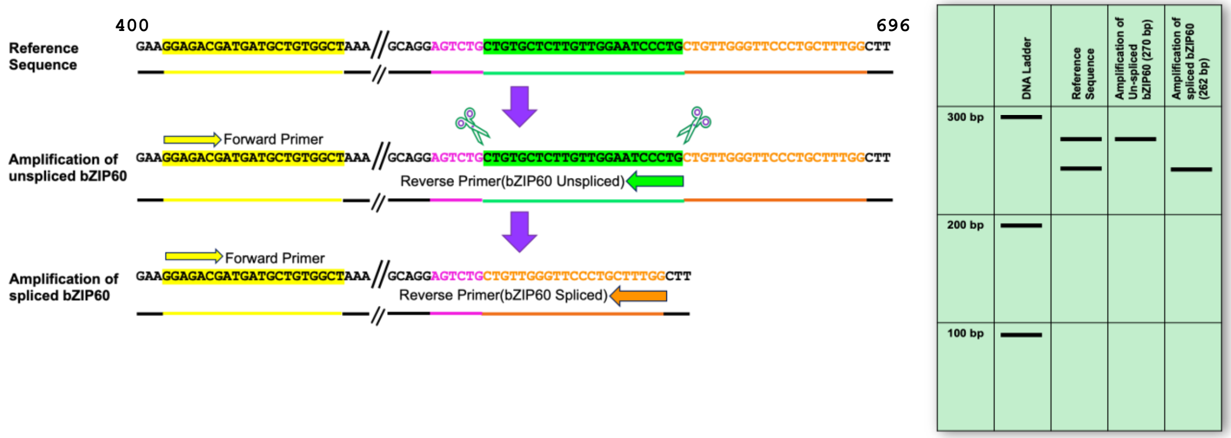

Supplementary Table S1. Primers used in this study.

Application	Gene	Sequence	Fwd/Rev
clone (attB flanked)	*IRE1a*	GGGG ACA AGT TTG TAC AAA AAA GCA GGC TCC GCAAGATAATATTGCATACCTAAGA	F1
clone (attB flanked)	*IRE1a*	GGGG ACC ACT TTG TAC AAG AAA GCT GGG TC AATAGTAGTAAGAAGAAAAGATGGC	R1
clone (attB flanked)	*IRE1a*	GGGG ACA AGT TTG TAC AAA AAA GCA GGC TCC CCTAAGATCATTTACCTTGATCTCA	F2
clone (attB flanked)	*IRE1a*	GGGG ACC ACT TTG TAC AAG AAA GCT GGG TC TTAAACATGCAGAGGATGGAAGAAA	R2
clone (attB flanked)	*IRE1b*	GGGG ACA AGT TTG TAC AAA AAA GCA GGC TCC GACATCATGCATCATGTTGATACTC	F1
clone (attB flanked)	*IRE1b*	GGGG ACC ACT TTG TAC AAG AAA GCT GGG TC GAGATAGATTTGTCATAGAACTTGG	R1
clone (attB flanked)	*IRE1b*	GGGG ACA AGT TTG TAC AAA AAA GCA GGC TCC CCCAACAGAGACATCATGCATCATG	F2

clone (attB flanked)	*IRE1b*	GGGG ACC ACT TTG TAC AAG AAA GCT GGG TC CCGATCGGATTGAGAGATTTGATTG	R2
clone (attB flanked)	*IRE1a*	GGGGACCACTTTGTACAAGAAAGCTGGGTC AGGACATCTCGGCGGCATGGT	R3
clone (attB flanked)	*IRE1a*	GGGGACCACTTTGTACAAGAAAGCTGGGTC TAGTAAGAAGAAAAGATGGCGGAG	R4
clone (attB flanked)	*IRE1b*	GGGGACCACTTTGTACAAGAAAGCTGGGTC GATTGGAGATAGATTTGTCAT	R3
clone (attB flanked)	*IRE1b*	GGGGACCACTTTGTACAAGAAAGCTGGGTC GATCGGATTGAGAGATTTGAT	R4
clone (attB flanked)	*IRE1b*	GGGG ACC ACT TTG TAC AAG AAA GCT GGG TC AGATAGATTTGTCATAGAACTTGG	R5
clone (attB flanked)	*IRE1b*	GGGG ACC ACT TTG TAC AAG AAA GCT GGG TC GATCGGATTGAGAGATTTGATTG	R6
Bigdye	*IRE1a*	CTAACCAATTTTTGCAAGATA	F1
Bigdye	*IRE1a*	GAGACG ATTA GATC CGCC TAGC	R1
Bigdye	*IRE1a*	CGGTTCTATTAATCAGTGAG	F2
Bigdye	*IRE1a*	CATATA CATT AGAC ATAG TGGC	R2
Bigdye	*IRE1a*	GCGCTAAGCGGAGGGTCACCGC	F3
Bigdye	*IRE1a*	CGTTC TCCT TTTG AATC TTCT	R3
Bigdye	*IRE1a*	ACGCATATTGGCTCGGCTCGGC	F4
Bigdye	*IRE1a*	TATGG AGAA TCCG AACA GAGA	R4
Bigdye	*IRE1a*	CTCGCCGGACAGAGACGGAGA	F5
Bigdye	*IRE1a*	AACTAA GTAT TTTT AAAC ATGC	R5
Bigdye	*IRE1b*	AAACGTTATATACAAGGTCCGTT	F1
Bigdye	*IRE1b*	GTG TGA CAA TTT CTA ATT GAC CAC	R1
Bigdye	*IRE1b*	AATTAGAAATTGTCACACGTCTCTA	F2
Bigdye	*IRE1b*	AT TAT ATG TTG GTT TAG GCA AAC	R2
Bigdye	*IRE1b*	AGAATATGTTTGCCTAAACCAAC	F3
Bigdye	*IRE1b*	TT AAA GAT GTT TTT GTT TGT TTG	R3
Bigdye	*IRE1b*	AATCACCGATTTAAACCGATAA	F4
Bigdye	*IRE1b*	AA TGT CAA ATG AAT CAA ACG AAA	R4
Bigdye	*IRE1a*	GCAAGATAATATTGCATACCTAAGA	F6a
Bigdye	*IRE1a*	CCTTTTGCTATCAACAAATT	R6a
Bigdye	*IRE1a*	GCCACTATGTCTAATGTATATGTG	F7a
Bigdye	*IRE1a*	TAGTAAGAAGAAAAGATGGCG	R7a
Bigdye	*IRE1b*	ATCATGCATCATGTTGATACT	F5b
Bigdye	*IRE1b*	AGTTGTTGAGATTTTTTTTCC	R5b
Bigdye	*IRE1b*	CCAAATTTGGATGACACTTAT	F6b
Bigdye	*IRE1b*	ATAACAAACAATAACCGGATC	R6b

Bigdye	*IRE1b*	AACCAATGATTTACCAAATTG	F7b
Bigdye	*IRE1b*	AGATAGATTTGTCATAGAACTTGG	R7b
Bigdye	*pDNOR207*	TCGCGTTAACGCTAGCATGGATCTC	F
Bigdye	*pDNOR207*	GTAACATCAGAGATTTTGAGACAC	R
Bigdye	*GUS*	CGGTGAACAG GTATGGAATT TCGC	F
Bigdye	*GUS*	TTCCCACCAA CGCTGATCAA TTCC	R
qPCR	*IRE1a F*	GCTTCAGACCTCATATCCCG	F
qPCR	*IRE1a R*	AGCATCACGAAGGAAAGACAG	R
qPCR	*IRE1b F*	GGTGGGATGAGAAACTGGATAG	F
qPCR	*IRE1b R*	AGTTTGTTCCGTATGACCCG	R
qPCR	*UBQ5 F*	GACGCTTCATCTCGTCC	F
qPCR	*UBQ5 R*	GTAAACGTAGGTGAGTCCA	R
qPCR	*ERDJ3B*	CAAATACGAACGGGAGGGATAC	F
qPCR	*ERDJ3B*	GGTTCGCCGTCTTCATAGAAA	R
qPCR	*SDF2*	TCAAGAGTGGAGCAACCATTAG	F
qPCR	*SDF2*	CCAAAGCAGCTAACCTCTAAGT	R
qPCR	*ERdj3A*	GGTAGCTCATCGAATGCTGAA	F
qPCR	*ERdj3A*	GGTCCACAACGTCCTTCTTATAG	R
qPCR	*BIP3*	CGGTCCAAGGTGGAGTATTAAG	F
qPCR	*BIP3*	CGCCTCCGACAGTTTCAATA	R
qPCR	*BIP1/2*	CTGCTGTTCAGGGTGGTATT	F
qPCR	*BIP1/2*	TCATCACTCCTCCTACAGTCTC	R
qPCR	*bZIP60us_FWD*	GGAGACGATGATGCTGTGGCT	F
qPCR	*bZIP60u_REV*	CAGGGATTCCAACAAGAGCACAG	R
qPCR	*bZIP60s_REV*	CAGGGAACCCAACAGCAGACT	R
